# A scoping review of influenza RdRp-targeting inhibitors: mechanisms, clinical translation, and emerging challenges

**DOI:** 10.3389/fmicb.2026.1860682

**Published:** 2026-06-26

**Authors:** Ying Chen, Sibo Wang, Anqi Feng, Ruoya Qian, Dong Wei, Erzhen Chen, Zhitao Yang

**Affiliations:** 1 Department of Emergency, Ruijin Hospital, Shanghai Jiao Tong University School of Medicine, Shanghai, China; 2 Research Laboratory of Clinical Virology, Department of Infectious Diseases, Ruijin Hospital, Shanghai Jiao Tong University School of Medicine, Shanghai, China; 3 Shanghai Institute of Aviation Medicine, Ruijin Hospital, Shanghai Jiao Tong University School of Medicine, Shanghai, China; 4 Department of Emergency, Ruijin Hospital Taicang, Jiangsu, China

**Keywords:** antiviral resistance, influenza, PA endonuclease inhibitors, PB inhibitors, RdRp-targeting inhibitors, RNA polymerase inhibitors

## Abstract

**Background:**

Although the RNA-dependent RNA polymerase (RdRp) complex is a therapeutic target for influenza, evidence on the pharmacology, resistance, and clinical impact of RdRp-targeting inhibitors in high-risk populations remains fragmented. This scoping review on RdRp-targeting inhibitors identifies the research gaps and characterizes their mechanisms of action, pharmacokinetics, efficacy, safety, and resistance patterns.

**Methods:**

Following the “Preferred Reporting Items for Systematic Reviews and Meta-Analyses extension for Scoping Reviews” guidelines, a comprehensive search for studies was conducted across PubMed, Embase, the Cochrane Library, China National Knowledge Infrastructure, Wanfang, and ClinicalTrials.gov from inception to October 2025. Preclinical and clinical studies on influenza RdRp-targeting inhibitors were included, with data charted across eight domains.

**Results:**

From 1,282 identified records (English: 1197; Chinese: 85), 156 articles were included. PA inhibitors emerged as the most extensively documented class. Preclinical findings demonstrated potent antiviral activity of PA inhibitors and emerging PB1/PB2 analogs, with variability in pharmacokinetic profiles. Clinical evidence showed PA inhibitors consistently shorten the time to symptom relief and accelerate viral RNA clearance compared with standard therapy. RdRp-targeting inhibitors showed an acceptable tolerability profile. Resistance was a notable challenge, primarily involving PA-I38 substitutions. Evidence on drug–drug interactions was limited to early-phase trials. A significant evidence gap remains for high-risk populations; baloxavir being the only agent widely studied in high-risk adults.

**Conclusion:**

RdRp-targeting antivirals represent a promising frontier for influenza treatment, with PA inhibitors being the most extensively validated class. While PB1 and PB2 inhibitors diversify the therapeutic pipeline, their development is hindered by the need for multiple-dose regimens and the emergence of drug resistance. Future research should prioritize inhibitor designs that minimize resistance, as well as combination regimens, to accelerate clinical translation.

## 1 Introduction

Influenza viruses cause seasonal epidemics and occasional pandemics, with an estimated 3 to 5 million severe cases and up to 650,000 deaths annually worldwide (WHO, 2025a, 2025b, 2025c). Influenza viruses primarily infect airway and alveolar epithelial cells via α2,6-linked sialic acid receptors, leading to epithelial damage and increased morbidity in individuals with chronic respiratory or cardiovascular disease, diabetes, and immunosuppression (Benam et al., 2019). East Asia experiences a particularly high influenza burden, influenced by factors such as limited public awareness, gaps in healthcare infrastructure, high population density, and frequent human-animal interactions (Haider and Hassan, 2025).

Antiviral therapy remains critical, particularly for high-risk patients, especially elderly and hospitalized patients. However, current treatment options have limitations (Bonomini et al., 2025; Kumari et al., 2025). M2 ion channel inhibitors such as amantadine and rimantadine are no longer recommended because of widespread resistance (>90% of circulating H3N2 isolates resistant) (De Clercq, 2006; Weinstock, 2006; Mtambo et al., 2021; CDC, 2024). Neuraminidase inhibitors (NAIs), including oseltamivir and zanamivir, remain the first-line therapy (Editors from The Medical Letter, 2024; CDC, 2025; WHO, 2025a); however, their clinical benefit in severe influenza is limited, and specific resistance mutations such as H275Y in N1 and E119V or R292K in N2 can significantly reduce efficacy (Burnham et al., 2013; Lee and Hurt, 2018; Omoto et al., 2018; Hanula et al., 2024).

Given these challenges, novel strategies targeting the viral RNA polymerase complex are under development. The vRNP complex comprises viral RNA, nucleoprotein (NP), and the heterotrimeric RNA-dependent RNA polymerase (RdRp) complex, which consists of the PA, PB1, and PB2 subunits. Within this assembly, the RdRp complex is essential for viral replication and transcription, making it an attractive antiviral target (Stevaert and Naesens, 2016). Although NP-directed compounds have also shown antiviral potential in preclinical studies, clinical and translational development has been most advanced for inhibitors targeting the polymerase subunits. Accordingly, this review focuses specifically on RdRp-targeting inhibitors. Based on subunit specificity, these inhibitors can be broadly classified into three categories: PA inhibitors such as baloxavir marboxil (BXM), which block cap-snatching and inhibit viral transcription; PB1 inhibitors (e.g., favipiravir), which impair viral RNA synthesis through lethal mutagenesis (Baranovich et al., 2013; Pflug et al., 2017; Bonomini et al., 2025); and PB2 inhibitors (e.g., pimodivir), which inhibit cap-dependent initiation of viral transcription (Omoto et al., 2018; Todd et al., 2021). Collectively, these agents disrupt polymerase function through distinct, subunit-specific mechanisms, offering the potential to overcome limitations of existing antiviral classes (Biswas et al., 1998; Davis et al., 2014; te Velthuis and Fodor, 2016; Takashita, 2021; Hou et al., 2022; Li J. et al., 2025).

Despite this therapeutic promise, the evidence base for RdRp-targeting inhibitors remains fragmented. In addition, the clinical utility of RdRp-targeting inhibitors is challenged by the emergence of resistant variants (Gubareva and Fry, 2019). While susceptibility of the infecting influenza virus is an important determinant of antiviral effectiveness, it is not the sole factor influencing resistance dynamics. The potential for resistant strains to emerge and circulate, even in the absence of drug pressure, raises significant public health concerns (Gubareva and Fry, 2019). Evidence from past influenza seasons and experimental studies suggests that resistant variants may retain transmissibility, underscoring the risk of community spread (Meijer et al., 2009). These observations highlight the need for global monitoring to detect resistance early, understand its epidemiological impact, and guide the development of next-generation antivirals capable of maintaining efficacy across diverse viral backgrounds. However, comprehensive data on structural optimization, molecular mechanisms, pharmacokinetic profiles, clinical efficacy, safety, drug–drug interaction potential, and resistance mechanisms are limited. Accordingly, this scoping review aims to map the existing literature on influenza RdRp-targeting inhibitors, identify research gaps, support informed drug development, and assess the translational value in addressing the global burden of influenza.

## 2 Methods

### 2.1 Review framework and objectives

This scoping review was conducted in accordance with the Preferred Reporting Items for Systematic Reviews and Meta-Analyses extension for Scoping Reviews (PRISMA-ScR) guidelines (Tricco et al., 2018). A completed PRISMA-ScR checklist is provided in the Supplementary material. A protocol was not registered for this scoping review.

### 2.2 Research questions

The review is structured around eight research questions designed to map the current evidence and highlight knowledge gaps related to RdRp-targeting inhibitors: (i) recent advancements in the mechanisms of action, core targets (PA, PB1, and PB2), and their implications on dosing strategies; (ii) findings from preclinical studies, including structural modifications and subunit-specific antiviral activity; (iii) pharmacokinetics (PK) properties of these agents and their implications for clinical use; (iv) reported efficacy of single-dose PA inhibitors versus multi-dose regimens for rapid symptom relief and viral clearance (viral RNA reduction, RNA-negative conversion, and changes in viral titers); (v) clinical outcomes with respect to safety and patient compliance; (vi) safety profiles in high-risk populations; (vii) resistance mechanisms across PA, PB1, and PB2 inhibitors, and, (viii) available evidence on drug–drug interactions, emphasizing the role of metabolic pathways, dosing strategies, and clinical safety in polypharmacy settings.

### 2.3 Literature search strategy

A comprehensive search was conducted across PubMed, Embase, Cochrane Library, ClinicalTrials.gov, and Chinese databases, including China National Knowledge Infrastructure and Wanfang, from inception till October 2025. Chinese-language databases were included to ensure capture of the global evidence base, particularly because several influenza polymerase-targeting antivirals identified in this review have been developed, clinically evaluated, or advanced through regulatory pathways in China. Inclusion of these sources was intended to reduce incomplete evidence capture and to improve representation of region-specific translational data within the overall synthesis.

Search algorithms incorporated controlled vocabulary and keywords related to influenza, RdRp-targeting inhibitors, and subunit-specific targets. The PubMed and Embase search strings were revised to correct terms with spelling errors or terms that did not retrieve results in PubMed. Abstracts and conference posters were also screened to capture emerging data. The following search terms were used: influenza, vRNP, viral ribonucleoprotein, PB1 subunit, PB2 subunit, PA subunit, inhibitor, antiviral, clinical, preclinical, mechanism, resistance, sebaloxavir marboxil, ZX-7101A, favipiravir, T-705, favipira, favilavir, abigan, avifavir, areplivir, J05AX27, onradivir, ZSP1273, ZSP-1273, baloxavir marboxil, xofluza, J05AX25, BXM (S-033188), S-033188, S-033447, BXA (S-033447), ADC189, ADC-189, deunoxavir marboxil, TG1000, TG-1000, pixavir marboxil, suraxavir marboxil, GP681, mabaloxavir, masulaxavir, and maseloxavir. A detailed search string has been provided in the Supplementary material.

### 2.4 Selection criteria

Eligible studies included adult participants aged>18 years, without restrictions on gender or race. The target population comprised individuals receiving treatment for Influenza A (subtypes H1N1 and H3N2) and influenza B viruses, with particular emphasis on high-risk and special populations, such as the elderly, pregnant women, patients with renal impairment, and immunocompromised individuals. The review included a range of study designs, including randomized clinical trials, observational studies, and preclinical investigations involving *in vitro* and *in vivo* models relevant to influenza RdRp-targeting inhibitor research. For this review, eligible interventions were restricted to polymerase-directed agents targeting the PA, PB1, or PB2 subunits for which data were available on one or more prespecified domains, including mechanism of action, PK, efficacy, safety, resistance, or drug–drug interaction potential.

Studies were excluded if they were case reports, post-hoc analyses, review articles, systematic reviews, meta-analyses, cost-effectiveness studies, study protocols, short communications, or brief communications. Duplicate publications, involving the same patient population, were removed to avoid data redundancy. Articles that did not align with the predefined objectives of the review were also omitted. Furthermore, studies focusing on non-influenza viruses, such as coronavirus or respiratory syncytial virus, were excluded. Research investigating non-drug interventions, including vaccines, traditional Chinese medicine, dietary supplements, physical therapies, or drugs not relevant to influenza treatment (e.g., antibiotics) were excluded. Studies evaluating antivirals directed primarily against NP, hemagglutinin, neuraminidase, or host targets were excluded from the present review. In addition, agents designed to disrupt PB1–PA or PB1–PB2 protein–protein interfaces, including peptide-based inhibitors, were not included because these approaches remain largely exploratory and lacked sufficient translationally comparable evidence across the review domains prespecified for evidence charting.

### 2.5 Screening and eligibility assessment

All retrieved records were managed in a master database. Two independent reviewers screened titles and abstracts for relevance. Full texts of potentially eligible studies were reviewed independently using a standardized checklist. Discrepancies were resolved by consensus or adjudicated by a third reviewer.

### 2.6 Data extraction

A structured data extraction form was developed in Microsoft Excel and piloted on 5 studies to ensure clarity and consistency. Extracted data included study characteristics, RdRp subunit target, mechanism of action, dosing regimens, PK parameters, clinical outcomes, safety profiles, virological endpoints, and resistance data. Two reviewers independently extracted and cross-validated data to ensure accuracy. Discrepancies were resolved by consensus or adjudicated by a third reviewer. Formal critical appraisal of individual sources was not conducted, consistent with the exploratory evidence-mapping purpose of this scoping review.

## 3 Results

### 3.1 Overview of search results and study characteristics

**Figure 1:**
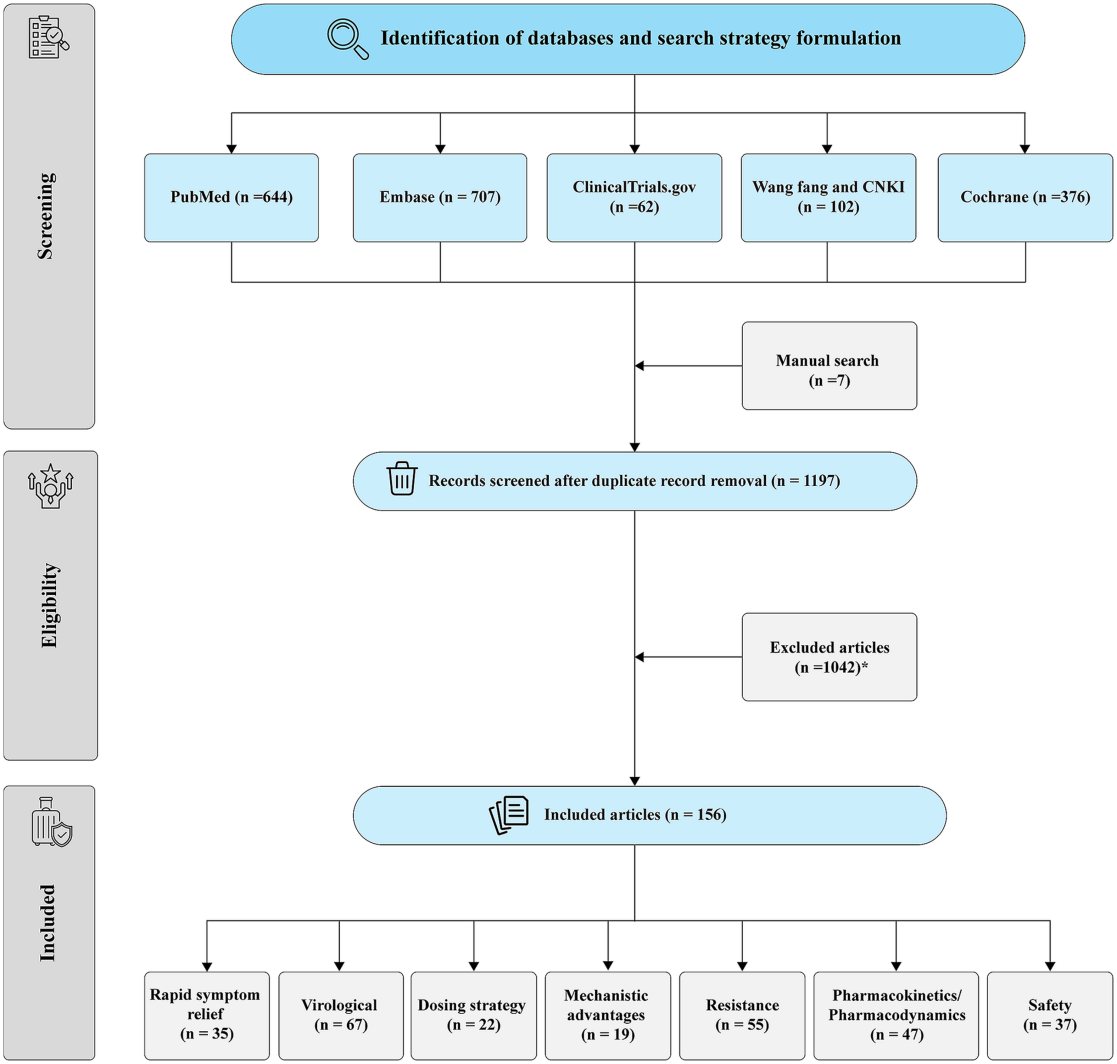
Flow diagram depicting the flow of information through different phases of a scoping review. *The studies not related to influenza RdRp-targeting inhibitors, non-drug interventions, participants <18 years, non-influenza viruses, case reports, *post-hoc* analyses, reviews/meta-analyses, or study protocols/short communications/duplicates.

The literature search initially identified 1,282 articles, including 1,197 English and 85 Chinese publications. After removing duplicates and applying the predefined inclusion and exclusion criteria, 156 studies were included in the final analysis. The study selection process is illustrated in Figure 1, following the PRISMA-ScR framework.

### 3.2 Evidence across key domains

The included studies collectively provide a comprehensive overview of influenza RdRp-targeting inhibitors across multiple domains.

Nineteen studies reported antiviral activities and mechanisms of RdRp-targeting inhibitors, highlighting the roles of PA, PB1, and PB2 subunits in viral replication (Furuta et al., 2005; Kiso et al., 2010; Baranovich et al., 2013; Jin et al., 2013; Sangawa et al., 2013; Han et al., 2018; Noshi et al., 2018; Omoto et al., 2018; Pagadala, 2019; Kumar et al., 2021; Todd et al., 2021; Wang et al., 2021; Retout et al., 2022; Saim-Mamoun et al., 2022; Chen et al., 2023, 2024a; Kohlbrand et al., 2024; Fage et al., 2025).

A total of 55 studies reported resistance mechanisms, indicating variability in the emergence and the molecular basis of resistance across RdRp-targeting subunits (Kiso et al., 2010; Sleeman et al., 2010; Baranovich et al., 2013; Naesens et al., 2013; Smee et al., 2013; Cheung et al., 2014; Zhang et al., 2014; Marathe et al., 2016; Ormond et al., 2017; Baz et al., 2018; Goldhill et al., 2018; Han et al., 2018; Kiso et al., 2018; Noshi et al., 2018; Fukao et al., 2019b; Goldhill et al., 2019; Gubareva et al., 2019; Pascua et al., 2019; Yoshino et al., 2019; Ikematsu et al., 2020b; Ison et al., 2020; Umemura et al., 2020; Wang et al., 2020a; Chong et al., 2021; Goldhill et al., 2021; Hamza et al., 2021; Jones et al., 2021; Kumar et al., 2021; Lee et al., 2021, 2021; Suzuki et al., 2021; Todd et al., 2021; Atim et al., 2022; Hickerson et al., 2022; Jones et al., 2022; Komeno et al., 2022; Kumar et al., 2022; Miyazawa et al., 2022; Stannard et al., 2022; Kiso et al., 2023; Luo et al., 2023; Mu et al., 2023; Oh et al., 2023; Acocal-Juárez et al., 2024; Guo et al., 2024; Jin et al., 2024; Kohlbrand et al., 2024; Qiu et al., 2024; Ren et al., 2024; Taniguchi et al., 2024; Monto et al., 2025; Takashita et al., 2025; Wang H. et al., 2025).

Forty-seven studies reported PK/PD data, showing that absorption, half-life, and bioavailability significantly influence therapeutic efficacy and dosing schedules (Furuta et al., 2002; Smee et al., 2009, 2010; L’Huillier et al., 2015; Kawasaki et al., 2016; Takashita et al., 2016; Han et al., 2018; Kawaguchi et al., 2018b; Koshimichi et al., 2018; Trevejo et al., 2018; Fukao et al., 2019a; Pagadala, 2019; Watanabe et al., 2019; Kitano et al., 2020; Takashita et al., 2020; Twabela et al., 2020; Ando et al., 2021; Hu et al., 2021; Park et al., 2021; Pascua et al., 2021; Rogolino et al., 2021; Hayden et al., 2022; Jones et al., 2022; Kim et al., 2022; Liu et al., 2022; Retout et al., 2022; Chen et al., 2023; Kuroda et al., 2023; Luo et al., 2023; Takashita et al., 2023; Xu et al., 2023; Bonomini et al., 2024a, 2024b; Chen et al., 2024a, 2024b; Han et al., 2024; Hayden et al., 2024; Liu et al., 2024; Ren et al., 2024; Li C. et al., 2025; Li H. et al., 2025; Pang et al., 2025; Wei et al., 2025; Wu et al., 2025b).

Thirty-five studies noted that single-dose PA inhibitors were most frequently associated with accelerated clinical improvement (MDVI, LLC, 2015; Kawaguchi et al., 2018b; Finberg et al., 2019; Shah et al., 2019; Shionogi, 2019; Watanabe et al., 2019; Yoshino et al., 2019; Fujita et al., 2020; Ison et al., 2020; Shah et al., 2020; Uehara et al., 2020; Yoshii et al., 2020; Yoshimura et al., 2020; Yoshino et al., 2020; Chong et al., 2021; Ison et al., 2021; Cagas et al., 2022; Collins et al., 2022; Hayden et al., 2022; Kumar et al., 2022; Liu et al., 2022; Retout et al., 2022; Liao et al., 2023; Cai et al., 2024; Goto et al., 2024; Qiu et al., 2024; Ringer et al., 2024; Yang et al., 2024; Jiang et al., 2025; Wang H. et al., 2025; Wang et al., 2025a; Yang et al., 2025; Zhao et al., 2025).

Virological outcomes from 67 studies demonstrated reductions in viral RNA, shorter RNA-negative conversion times, and decreased viral titers when compared with NAIs/placebo (Takahashi et al., 2003; Sidwell et al., 2007; Itoh et al., 2009; Smee et al., 2009, 2013; Kiso et al., 2010, 2018, 2019, 2020b, 2020a, 2023; Sleeman et al., 2010; Tarbet et al., 2012, 2014; Baranovich et al., 2013; Park et al., 2014, 2021; Vanderlinden et al., 2016; Imai et al., 2017; Kawaguchi et al., 2018a; Omoto et al., 2018; Trevejo et al., 2018; Mishin et al., 2019; Shionogi, 2019; Taniguchi et al., 2019; Watanabe et al., 2019; Abed et al., 2020; Baba et al., 2020; Checkmahomed et al., 2020; Chesnokov et al., 2020; Fang et al., 2020; Ison et al., 2020, 2021; Kitano et al., 2020; Koshimichi et al., 2020; Lee et al., 2020, 2021; Twabela et al., 2020; Uehara et al., 2020; Ando et al., 2021; Chong et al., 2021; Hamza et al., 2021; Pascua et al., 2021; Suzuki et al., 2021; Hayden et al., 2022, 2024; Jones et al., 2022; Kumar et al., 2022; Saim-Mamoun et al., 2022; Chen et al., 2023, 2024a; Kuroda et al., 2023; Li et al., 2023; Bonomini et al., 2024a; Goto et al., 2024; Jin et al., 2024; Jiang et al., 2025; Monto et al., 2025; Wang et al., 2025a; Wei et al., 2025; Yang et al., 2025; Zhao et al., 2025). Dosing strategies were reported in 22 studies, highlighting variability in optimal regimens depending on subunit specificity, PK profiles, and patient characteristics (Takahashi et al., 2003; Tarbet et al., 2012; MDVI, LLC, 2015; Kawaguchi et al., 2018b; Finberg et al., 2019; Fukao et al., 2019a; Kiso et al., 2019; Fang et al., 2020; Ikematsu et al., 2020a; Kitano et al., 2020; Twabela et al., 2020; Wang et al., 2020b, 2025b; Yoshino et al., 2020; Ando et al., 2021; Hu et al., 2021; Hayden et al., 2022, 2024; Chen et al., 2023; Kuroda et al., 2023; Pang et al., 2025; Wei et al., 2025).

Thirty-seven studies reported generally favorable safety profiles, though adverse events (AEs) were more frequent in high-risk populations (MDVI, LLC, 2015; Kawaguchi et al., 2018b; Koshimichi et al., 2018; Trevejo et al., 2018; Finberg et al., 2019; Shionogi, 2019; Ikematsu et al., 2020a; Ison et al., 2020; Wang et al., 2020b; Yoshino et al., 2020; Hu et al., 2021; Tanaka et al., 2021; Hara et al., 2022; Kim et al., 2022; Kumar et al., 2022; Liu et al., 2022; Luo et al., 2023; Ortiz et al., 2023; Xu et al., 2023; Cai et al., 2024; Han et al., 2024; Jin et al., 2024; Qiu et al., 2024; Yang et al., 2024; Zhou et al., 2024; Li C. et al., 2025; Li H. et al., 2025; Negru et al., 2025; Pang et al., 2025; Tu et al., 2025; Wang et al., 2025a; Wang H. et al., 2025; Wei et al., 2025; Wu et al., 2025b; Yang et al., 2025; Zhao et al., 2025).

### 3.3 Molecular mechanisms and drug target potential of Influenza polymerase subunits

Evidence from mechanistic research highlights the distinct functional roles of the PA, PB1, and PB2 subunits in viral RNA transcription and replication, including their coordinated involvement in the cap-snatching process, thereby providing multiple targets for selective antiviral intervention.

The PA subunit, particularly its N-terminal endonuclease (PAN) domain, mediates the cap-snatching process. This is an essential mechanism through which the virus cleaves host pre-mRNAs to generate capped RNA fragments that serve as primers for viral mRNA synthesis (Kumar et al., 2021; Hou et al., 2022). The PB1 subunit forms the catalytic core of the RNA-dependent RNA polymerase (RdRp) complex, directing the template elongation of nascent viral RNA by catalyzing the polymerization of ribonucleotides during both transcription and replication to ensure accurate genome synthesis. The PB2 subunit mediates cap-binding by recognizing the 5’cap structures of host mRNAs and initiates transcription (Kobayashi et al., 1996). Together, these three subunits orchestrate viral mRNA synthesis, with each representing a distinct and druggable antiviral target (Supplementary Figure 1 and Table 1). However, despite these mechanistic insights, comparative evaluations across subunits and their clinical implications remain insufficiently explored.

**Table 1:** Mechanistic overview of Influenza RdRp-targeting inhibitors.

Inhibitor	Target subunit	Mechanism of action	Binding site/domain	Key mechanistic feature/functional outcome	References
BXM/Baloxavir acid (BXA)	PA (PAN domain)	Cap-dependent endonuclease inhibition	PA endonuclease domain	Blocks host mRNA cleavage and cap-snatching; halts viral mRNA synthesis	Retout et al. (2022), Omoto et al. (2018), Saim-Mamoun et al. (2022), Noshi et al. (2018), Fage et al. (2025), Kumar et al. (2021), Kohlbrand et al. (2024), and Todd et al. (2021), Chen et al. (2024a)
Suraxavir marboxil (GP681)					
Sebaloxavir marboxil (ZX-7101A)					
Deunoxavir marboxil (ADC189)					
Pixavir marboxil (TG-1000)					
Tivoxavir marboxil					
ATV2301					
Compound 3 (novel)			Regions B, C, D of PA binding pocket	Avoids resistance mutation sites (e.g., I38), retains efficacy against drug-resistant strains	Ren et al. (2024)
ATV03	PA, RdRp, NP	Inhibits PA endonuclease; interferes with RdRp and NP	PA endonuclease domain	Blocks multiple RdRp functions; reduces viral replication	Chen et al. (2024b)
ATV07					
Favipiravir	PB1 (RdRp)	RNA-dependent RNA polymerase inhibition; purine analog; lethal mutagenesis; delayed chain termination	PB1 catalytic site; interacts with nascent RNA and template strand	Incorporates into viral RNA, induces G → A/C → T transitions; halts elongation; reduces viral infectivity	Komeno et al. (2022), Sidwell et al. (2007), Goldhill et al. (2021), Furuta et al. (2005), Sangawa et al. (2013), Han et al. (2018, p. 705), Kiso et al. (2010), Baranovich et al. (2013), Jin et al. (2013), and Wang et al. (2021)
AZT-TP	PB1	Nucleotide incorporation inhibitor	PB1 catalytic site near tip of priming loop	Prevents incorporation of incoming nucleotides; halts initiation of vRNA replication	Pagadala (2019)
Onradivir (ZSP1273)	PB2	Blocks cap-binding	PB2 cap-binding domain	By blocking cap binding, prevents effective initiation of viral mRNA synthesis, which disrupts the production of new viral RNA	Chen et al. (2023)
Pimodivir					Finberg et al. (2019)

ADME, Absorption, Distribution, Metabolism, and Excretion; ATV03, ATV07, ATV2301, compound codes; BXA, Baloxavir Acid; BXM, Baloxavir Marboxil; I38, Isoleucine 38; PA, residue in endonuclease domain; PB1, Polymerase Basic Protein 1; PB2, Polymerase Basic Protein 2; PA, Polymerase Acidic Protein; PAN, PA N-terminal domain; RdRp, RNA-dependent RNA polymerase.

### 3.4 Mechanism of action

#### 3.4.1 PA inhibitors

Preclinical and clinical studies consistently report that PA inhibitors act by binding to the endonuclease active site and chelating the essential divalent metal ions Mn2 + and Mg2+, thereby preventing cleavage of host pre-messenger RNAs (pre-mRNAs) and blocking the cap-snatching required for viral protein synthesis (DuBois et al., 2012; Takashita, 2021). The reported drugs in this class include baloxavir (BXM), suraxavir marboxil (GP681), tivoxavir marboxil, ATV2301, ATV03, and ATV07, deunoxavir marboxil (ADC189), sebaloxavir marboxil (ZX-7101A), and pixavir marboxil (TG-1000) (DuBois et al., 2012; Takashita, 2021).

#### 3.4.2 PB1 inhibitors

Favipiravir (T-705), the most extensively studied compound, acts as a nucleoside analog that is converted intracellularly into its active ribofuranosyl triphosphate metabolites (T-705RTP, Supplementary Figure 2I). These active metabolites are incorporated into nascent viral RNA, inducing lethal mutagenesis or premature chain termination, primarily through G-to-A and C-to-U transition mutations, which impairs the replication fidelity and produces non-viable viral genome (Wang et al., 2021; Komeno et al., 2022). Similarly, 3′-Azido-3′-deoxy-thymidine-5′-triphosphate (AZT-TP) targets the PB1 priming loop, interfering with the initiation phase of RNA synthesis and preventing elongation of the growing RNA chain (Supplementary Figure 2J) (Pagadala, 2019). Current evidence highlights these mechanisms as central to PB1-directed antiviral activity, though comparative data across analogs remain limited.

#### 3.4.3 PB2 inhibitors

Evidence indicates that PB2 inhibitors such as pimodivir competitively occupy the PB2 cap-binding site, preventing interaction with the 5′ cap structure (m^7^GTP) of host mRNA and thereby blocking cap-dependent transcription initiation. Secondly, pimodivir stabilizes an inactive “apo” conformation of the polymerase by interacting with the midlink domain, which perturbs the conformational equilibrium and suppresses polymerase activity. Structural studies show that these interactions involve hydrogen bonding, *π*–π stacking, and salt-bridge formation within the PB2 cap-binding and midlink domains. This dual mechanism distinguishes pimodivir from other inhibitors by combining competitive binding with conformational stabilization (Byrn et al., 2015; Chen et al., 2023; Li Y. et al., 2025).

### 3.5 Structural optimization

#### 3.5.1 PA inhibitors

The structural optimization of PA endonuclease inhibitors has largely focused on balancing three requirements: preservation of the metal-chelating pharmacophore, sufficient lipophilicity for oral exposure, and robust hydrophobic-pocket engagement to reduce susceptibility to resistance-associated substitutions. Most PA inhibitors contain a polar metal-chelating head group and a hydrophobic aromatic tail (Supplementary Figure 2). The polar pharmacophore, commonly represented by hydroxypyridone/diketo-acid-like motifs, anchors the inhibitor to the two catalytic divalent metal ions in the PA endonuclease active site and is therefore essential for target engagement (Todd et al., 2021). In contrast, the hydrophobic aromatic region occupies adjacent lipophilic pockets and stabilizes binding through van der Waals and hydrophobic interactions. This amphipathic architecture underlies the pharmacological profile of BXA, the active metabolite of BXM (Omoto et al., 2018; Dufrasne, 2021; de Souza et al., 2025).

BXM was developed as a prodrug to overcome the limited intestinal permeability of BXA, whose acidic keto–enol and metal-chelating structure increases polarity at physiological pH (Shishido et al., 2017; Omoto et al., 2018; Dufrasne, 2021). After oral administration, BXM is rapidly hydrolyzed to BXA, which chelates the catalytic metal ions in the cap-dependent endonuclease (CEN) active site and blocks host pre-mRNA cleavage (Supplementary Figure 2A) (Li J. et al., 2025). Structural studies indicate that the hydrophobic tail of BXA forms van der Waals contacts with residues such as Ala20, Tyr24, Lys34, Ala37, and Ile38 within the PA endonuclease binding pocket (Omoto et al., 2018; Dufrasne, 2021). This binding is possible since the part of BXA facing the hydrophobic binding pocket contains only few polar substituents (F atoms) (Omoto et al., 2018; Dufrasne, 2021). Accordingly, resistance-associated PA-I38 substitutions reduce BXA susceptibility largely by altering the size, polarity, and geometry of this hydrophobic pocket. For example, replacement of hydrophobic Ile38 by the more polar Thr residue weakens local van der Waals interactions and reduces complementarity between the inhibitor tail and the binding site. (Shishido et al., 2017; Omoto et al., 2018; Dufrasne, 2021; He et al., 2025). Therefore, next-generation PA inhibitors have attempted either to improve peripheral physicochemical properties or to reinforce the hydrophobic interaction network implicated in resistance (Shishido et al., 2017; Omoto et al., 2018; Dufrasne, 2021; He et al., 2025).

GP681 illustrates a peripheral heterocyclic optimization strategy. Compared with BXM, GP681 replaces the morpholine ring with a 4-oxa-7-azaspiro[2.5]octane moiety (Li J. et al., 2025), This spirocyclic replacement increases local hydrophobicity and conformational constraint around the polar heterocyclic region, which may improve hydrophilic–lipophilic balance, membrane permeability, and systemic exposure (Supplementary Figure 2B) (Wang et al., 2025b) However, because this modification is located outside the principal dibenzothiepin hydrophobic tail, its contribution to restoring hydrophobic interactions disrupted by PA-I38 substitutions is likely indirect. In addition, GP681 undergoes CYP3A4-mediated oxidative metabolism, indicating that this peripheral structural optimization, while improving local hydrophobicity and conformational rigidity, may introduce enzyme-dependent pharmacokinetic variability and potential drug–drug interaction concerns in polypharmacy settings (Li J. et al., 2025; Wang et al., 2025b).

ZX-7101A represents a different strategy by directly modifying the hydrophobic binding element. Independent medicinal chemistry analyses have established that the nature of the bulky hydrophobic group (BHG) is a critical determinant of PA endonuclease inhibitor potency across the BXM analogue series (Xu et al., 2023). ZX-7101A incorporates selenium into the hydrophobic dibenzothiepin-like scaffold, where the sulfur-to-selenium substitution increases ring rigidity and enhances van der Waals interactions at the binding interface (Xu et al., 2023). By directly reinforcing the hydrophobic interaction network — rather than relying on peripheral modifications — ZX-7101A provides a structurally grounded rationale for maintaining potency against PA-I38 resistant variants. This structural rationale is consistent with the favorable clinical resistance profile reported for ZX-7101A. In contrast to GP681, ZX-7101A exhibits limited CYP-mediated metabolism and a favorable drug–drug interaction profile based on available data (Supplementary Figures 2D,E).

Other PA inhibitors reflect additional optimization strategies. ADC189 uses deuteration to improve metabolic stability and systemic exposure without substantially changing the target-binding pharmacophore (Li J. et al., 2025; Zhao et al., 2025) (Supplementary Figure 2C). TG-1000 introduces a hydrophobic bicyclic moiety into the polar region, aiming to improve physicochemical balance and oral exposure while retaining PA endonuclease inhibition (DuBois et al., 2012; Takashita, 2021). Resistance-focused compounds, including ATV2301, ATV03, ATV07, and Compound 3, further extend this principle by combining preserved metal chelation with hydrophobic or heterocyclic substitutions that engage additional subpockets beyond the I38 region (Supplementary Figures 2F–H) (Chen et al., 2024b). Collectively, the structural evolution of PA inhibitors shows a consistent trend: the metal-chelating head group is conserved to maintain catalytic-site anchoring, whereas hydrophobic and peripheral substituents are fine-tuned to improve oral bioavailability, systemic exposure, binding robustness, and resistance tolerance.

#### 3.5.2 PB1 inhibitors

Favipiravir’s pyrazinecarboxamide scaffold has been the foundation for several optimization programs aimed at improving bioavailability, cellular uptake, and selectivity for viral polymerase over host polymerases (Konstantinova et al., 2022). Although major marketed structural derivatives are lacking, preclinical analogs involving nucleobase fluorination and amide chain modification improved polymerase selectivity and intracellular activation efficiency. For instance, synthetic analogs T-1105 and T-1106, have demonstrated substantial *in vitro* antiviral activity against influenza virus A/PR/8/34 (H1N1) (Furuta et al., 2009; Konstantinova et al., 2022). However, additional clinical studies are required to assess their potential for further development such as antiviral therapeutics.

#### 3.5.3 PB2 inhibitors

Early PB2 inhibitors such as pimodivir demonstrated potent, selective inhibition against influenza A but had limited efficacy against influenza B, susceptibility to specific resistance mutations, and reduced metabolic stability (Clark et al., 2014). Structural refinements focus on increasing broad-spectrum coverage, reducing cross-resistance, and achieving highly effective delivery into the lung and superior lung exposure (Cocrystal Pharma Inc, 2024, 2025). Optimization efforts led to the design of CC-42344, a novel PB2 inhibitor, featuring enhanced lipophilicity and improved binding to the PB2 cap-binding pocket (Cocrystal Pharma, Inc, 2024, 2025). Parallel scaffold optimization has also been applied to pimodivir and its successor onradivir (ZSP1273), which retains activity specifically against influenza A viruses. Structure-guided optimization of the pyridone and amide substituents strengthened hydrogen bonding with key residues, whereas aromatic ring expansion improved fit within the PB2 cap-binding pocket. These molecular refinements improved both target engagement and metabolic stability (Supplementary Figure 2K) (Hu et al., 2021; Chen et al., 2023).

### 3.6 Resistance mutations

#### 3.6.1 PA inhibitors

Resistance to influenza antivirals targeting the RdRp complex primarily arises from amino acid substitutions that alter polymerase conformation and reduce drug-binding affinity. Clinical trials and post-market surveillance indicate that the overall resistance rate to PA inhibitors ranges typically from 2 to 10% depending on viral subtype and treatment cohort (e.g., 2.2% in A[H1N1] and 9.7% in A[H3N2] for BXM-treated cases) (Jiang et al., 2025).

For PA endonuclease inhibitors, resistance is mainly associated with mutations at the I38 residue (e.g., I38T, I38M, and I38F), which reduce inhibitor binding to catalytic site while preserving enzymatic function (te Velthuis and Fodor, 2016). Structural analyses have shown that these substitutions disrupt hydrophobic and *π*–π stacking interactions within the catalytic pocket, thereby decreasing inhibitor potency and variably affecting polymerase activity (DuBois et al., 2012).

Of these, I38T is the most clinically relevant, which diminishes susceptibility to BXM and related compounds (Kuroda et al., 2023). In the CAPSTONE-1 trials, I38T/M/F variants emerged in 2.2 and 9.7% of BXM treated patients (Hayden et al., 2018), while CAPSTONE-2 reported similar substitutions in 5% of patients (Ison et al., 2020). These mutations significantly reduced BXM susceptibility *in vitro* and impacted virological outcomes.

In the FLAGSTONE trial conducted in hospitalized patients with severe influenza, combination therapy with BXM and oseltamivir lowered the incidence of treatment-emergent PA I38X-substitutions (2%), suggesting synergistic effects that mitigate resistance (Kumar et al., 2022).

Next,-generation PA inhibitors are being developed to improve resistance tolerance while preserving favorable pharmacokinetic and antiviral properties. Clinical data indicate a favorable resistance profile for ZX-7101A, with PA-I38T variants emerging in only approximately 1.8% of treated individuals, suggesting numerically lower observed PA-I38T emergence than the 2.2–9.7% rates reported in BXM-treated patients, although cross-trial comparisons should be interpreted cautiously (Wang H. et al., 2025). Low subtype-specific PA-I38T emergence rates were also reported for GP681 in a phase 3 trial (0.7% [1/138] in H1N1pdm and 0.9% [2/213] in H3N2). However, because the denominator definition and paired sequencing-evaluable population for GP681 were not fully transparent, these rates should be interpreted cautiously (Wang et al., 2025b).

Preclinical studies by Luo et al. (2023) identified PA-E18G as a novel substitution emerging after 15 serial passages of H1N1 in the presence of ZX-7101 in vitro. This substitution reduced susceptibility to both ZX-7101 and BXA; however, it should be interpreted strictly as an in vitro resistance finding, and its clinical relevance remains to be established (Luo et al., 2023).

Collectively, current evidence indicates that resistance-associated mutations to PA inhibitors are mechanistically well defined and detectable under therapeutic pressure, although their clinical impact varies by compound, viral subtype, host population, and treatment setting. Continuous genomic surveillance is nonetheless critical to monitor the emergence, prevalence, and fitness dynamics of these variants to preserve the long-term effectiveness of next-generation RdRp-targeting antivirals. (Supplementary Table 5).

#### 3.6.2 PB1 inhibitors

Resistance to favipiravir has been associated with K229R substitution in the PB1 active site and P653L in the PA subunit, which alter the interaction between the inhibitor and the nucleotide-binding pocket (Goldhill et al., 2021; Komeno et al., 2022).

Although K229R confers resistance, it also imposes a fitness cost by impairing polymerase activity (Goldhill et al., 2021). In contrast, modelling and *in vivo* studies have shown that the PA P653L mutation can provide a compensatory fitness advantage in certain viral backgrounds. Specifically, in early A(H1N1) pdm09 isolates such as A/England/195/2009, P653L improved polymerase fitness. This fitness gain enabled reassortment events in which the virus carrying both K229R and P653L preferentially acquired a wild-type PB1 segment, thereby losing the resistance-associated K229R mutation (Goldhill et al., 2021). Similarly, the PB1-V43I mutation in the polymerase complex of either H3N2 or H5N1 viruses has resulted in decreased susceptibility to favipiravir compared to wild type (Cheung et al., 2014).

On the other hand, viruses carrying the mutations H274Y (Kiso et al., 2010), N294S (Kiso et al., 2010), R292K (Zhang et al., 2014), and H275Y (Sleeman et al., 2010; Baz et al., 2018; Wang et al., 2020a) remained fully susceptible to favipiravir. Together, these findings underscore that favipiravir resistance dynamics depend strongly on viral genetic background. Continued surveillance, combined with molecular modelling, remains essential to predict resistance emergence and to inform the design of next-generation favipiravir analogs (Supplementary Table 5).

#### 3.6.3 PB2 inhibitors

Resistance mutations in PB2 primarily occur within the cap-binding domain.

Although early investigations of pimodivir identified substitutions such as F404Y, M431I, (Trevejo et al., 2018) and H357N (Gregor et al., 2021) that reduced inhibitor affinity while preserving cap-binding function, these data are of limited relevance as pimodivir development has since been discontinued.

In contrast, current efforts focus on ZSP1273. Preclinical studies have reported that ZSP1273 maintains antiviral activity against both oseltamivir-resistant and BXM-resistant (PA-I38T substitutions) influenza A strains, with EC50 values of 0.014 nM–0.017 nM, and 0.028 nM, respectively. At present, resistance associated with ZSP1273 has been characterized only in preclinical models, and no clinical resistance data are available. Continued monitoring during later-phase clinical development will be essential to determine the emergence and clinical relevance of PB2-targeted resistance (Chen et al., 2023) (Supplementary Table 5).

### 3.7 Preclinical findings

Preclinical studies have extensively evaluated RdRp-targeting inhibitors and reveal strong antiviral activity across PA, PB1, and PB2 targets, with PA inhibitors dominating the evidence landscape.

Among PA inhibitors, BXM has consistently exhibited potent antiviral activity in Madin–Darby Canine Kidney (MDCK) cells against (Abed et al., 2020) influenza A strains (IC_50_: 0.28 to 7.6 nM) and against influenza B (IC_50_: 2.43 to 3.42 nM) (Takashita et al., 2018). Significant viral titer reduction has also been observed in animal models, including full protection in avian studies (Twabela et al., 2020) and transmission prevention in ferrets (Lee et al., 2020, 2021) and mice (Kiso et al., 2020b; Kuroda et al., 2023). Similarly, ZX-7101A has demonstrated significant antiviral activity achieving EC_50_ values as low as 1.03 nM against H3N2 viruses and viral RNA load reduction in murine lungs (Luo et al., 2023). ATV-series compounds (ATV2301, ATV03, ATV07) have been reported to exhibit potency comparable to BXM, with EC₅₀ values reaching 0.78 nM for influenza A and 2.02–2.32 nM against influenza B viruses (Chen et al., 2024a, 2024b). TG-1000 has also been reported to exhibit in vitro activity against influenza A and B at nanomolar concentrations (0.35–3.10 nM and 2.75–11.90 nM, respectively). Preclinical studies in murine models indicated reductions in lung viral titers and improved survival rates compared with placebo (Xu et al., 2023).

Similarly, the PB1 inhibitor favipiravir showed broad-spectrum activity but at micromolar concentrations (IC₅₀: 0.14–0.99 μM) (Takashita et al., 2016); EC_50_: 4.50–7.49 μM against influenza B and 2.6–12.1 μM against influenza A viruses (Sidwell et al., 2007; Fang et al., 2020), indicating higher dosing requirements. PB2 inhibitor ZSP1273 demonstrated strong in vivo activity against influenza A, reducing lung viral titers by up to 4.97 log₁₀ at 120 h post-infection and achieving complete clearance by 192 h, with no antiviral activity against influenza B (Chen et al., 2023) (Supplementary Table 1).

Across these findings, PA inhibitors offer the most advanced preclinical profile and single-dose potential, whereas PB1 and PB2 inhibitors lack comparative depth in resistance characterization and pharmacokinetic optimization. Evidence gaps persist in head-to-head evaluations underscoring the need for systematic exploration of PB1 and PB2 inhibitors alongside integrated strategies.

### 3.8 Pharmacokinetics

The PK profiles of viral RdRp-targeting inhibitors demonstrate considerable variability, depending on the compound, dose, route of administration, and patient-related factors (Supplementary Table 2 and Table 2).

**Table 2:** Key pharmacokinetic and development characteristics of influenza polymerase-targeting inhibitors.

Drug candidate	Recommended dose (optimized)	Half-life (t_1/2_)	Primary route	Metabolism	Current status
BXM	40–80 mg (Single Dose) (US Food Drug Administration, 2024)	~49–91 h (US Food Drug Administration, 2024)	Oral (Li J. et al., 2025)	UGT1A3/CYP3A4 (Heo, 2018)	Approved in multiple countries (Li J. et al., 2025)
Suraxavir marboxil (GP681)	40 mg (Jiangxi Kerui Pharmaceuticals Co. Ltd, 2025)	65.8 h (Jiangxi Kerui Pharmaceuticals Co. Ltd, 2025)	Oral (Li J. et al., 2025)	(CYP) 3A4-mediated	Approved for marketing in China (Li J. et al., 2025)
Sebaloxavir marboxil (ZX-7101A)	40 or 80 mg (single dose) (Wu et al., 2025b)	102.3–125.55 h (Wu et al., 2025b)	Oral (Li J. et al., 2025)	UGT2B7 (Nanjing Zenshine Pharmaceuticals Co., Ltd, 2025)	Approved for marketing in China (Li J. et al., 2025)
Deunoxavir marboxil (ADC189)	45–90 mg (single dose) (Jiaxing Antigon Biotechnology Co. Ltd, 2024)	~ 76.69 to 98.28 h	Oral (Li J. et al., 2025)	NA	NDA (Li J. et al., 2025)
Pixavir marboxil (TG-1000)	40 or 80 mg (Xu et al., 2023)	~ 36 h (Xu et al., 2023)	Oral (Li J. et al., 2025)	The drug is rapidly converted to TG-0527 by hydrolysis, TG-0527 is eliminated primarily through hepatic metabolism (Xu et al., 2023)	NDA (Li J. et al., 2025)
Favipiravir	1800 mg BID on day 1, 800 mg BID on days 2–5 (Hayden et al., 2022)	~2–5 h	Oral (Li J. et al., 2025)	Aldehyde Oxidase (Wattana et al., 2023)	Approved for marketing in China (Li J. et al., 2025)
Onradivir (ZSP1273)	600 mg BID for 5 days (Yang et al., 2024)	~12.1 to 35.0 h (Li C. et al., 2025)	Oral (Li J. et al., 2025)	UDP-glucuronosyltransferases (Li C. et al., 2025)	Approved for marketing in China (Li J. et al., 2025)
Pimodivir	600 mg BID (Clinical Phase) (Deleu et al., 2018; Leopold et al., 2025)	~15–20 h	Oral (Li J. et al., 2025)	Minimal CYP involvement	Phase 3 clinical trials halted (Li J. et al., 2025)

NDA, New Drug Application.

Among the PA endonuclease inhibitors, BXM serves as the clinical benchmark, showing dose-dependent PK across 6 to 80 mg (Ando et al., 2021; Liu et al., 2022). The usual dosing regimen for BXM has been reported as a single oral dose of 40 mg for patients weighing 20–80 kg and 80 mg for those weighing >80 kg (US Food Drug Administration, 2024). It exhibits a prolonged elimination half-life (t_1/2_) of 48.9 to 114 h (Koshimichi et al., 2018), enabling sustained plasma concentrations above the inhibitory threshold for >5 days after a single dose (Kawaguchi et al., 2018b; Koshimichi et al., 2018; Ando et al., 2021; Kim et al., 2022; Liu et al., 2022; Pang et al., 2025). Similarly, ADC189 is reported to exhibit a dose-dependent PK across 15 to 90 mg, with a recommended single-dose regimen of 45 mg for individuals weighing 20–80 kg and 90 mg for those weighing > 80 kg. Its terminal t_1/2_ ranged from 76.69 to 98.28 h (Wei et al., 2025). GP681 exhibits a similar extended t_1/2_ of 65.8 ± 12.1 h, with a currently recommended dose of 40 mg. However, its cytochrome (CYP) 3A4-mediated metabolism introduces potential drug–drug interaction concerns (Jiangxi Qingfeng Pharmaceutical Co. Ltd, 2024). Of note, ZX-7101A achieves an extended t_1/2_ ranging from 83–126.4 h (Wu et al., 2025a), maintaining effective plasma levels beyond 144 h (Wu et al., 2025b), supporting single-dose therapy. Phase 1 studies evaluated 40 mg and 80 mg as single-dose regimens of ZX-7101A for adults; however, the approved adult dosage specified in the package insert is 80 mg (Uehara et al., 2020; Wu et al., 2025b). TG-1000 has demonstrated a relatively shorter t_1/2_ of 33 to 38 h across doses of 10 to 160 mg (Goto et al., 2024). The PK data also suggests nonlinear absorption characteristics of TG-1000 due to saturation effects. TG-1000 is typically administered as a single oral dose of 40 mg for patients weighing 40–80 kg and 80 mg for those weighing >80 kg. Following administration, TG-1000 undergoes hydrolysis to TG-0527 reaching its C_max_ within 3.5 h (Goto et al., 2024).

In the case of PB1 inhibitors, favipiravir, requires twice-daily dosing due to rapid clearance (t_½_: 2–5 h) despite high peak concentrations (Hayden et al., 2022). Its PK are complex, nonlinear, and dose-dependent, posing challenges for optimal dose determination. The initially approved regimen in Japan was 1,600 mg BID on day 1, followed by 600 mg BID thereafter. However, plasma concentrations in the United States participants were approximately 50% lower than those observed in Japan, raising concerns about the influence of body weight and ethnicity on favipiravir PK. PB2 inhibitors display intermediate profiles, with pimodivir displaying t_1/2_ ≈ 16 h (Trevejo et al., 2018) and ZSP1273 ranging from 12.08 to 34.98 h across 100 to 1,200 mg doses (Hu et al., 2021; Chen et al., 2023; Li C. et al., 2025; Li H. et al., 2025; Pang et al., 2025).

The key trends indicate that several PA inhibitors, particularly BXM, ADC189, GP681, and ZX-7101A, provide prolonged systemic exposure supporting simplified dosing. TG-1000 also supports single-dose administration but has a relatively shorter half-life than these longer-acting PA inhibitors. In contrast, PB1 and PB2 inhibitors are more often constrained by rapid clearance or multidose requirements. Evidence gaps persist in comparative PK/PD modeling, evaluation of metabolic variability in high-risk populations, and optimization of PB1 and PB2 inhibitors for extended coverage. These findings emphasize the translational advantage of PA inhibitors and the need for strategies that enhance pharmacokinetic durability across all RdRp-targeting inhibitor classes.

### 3.9 Clinical and virological efficacy

The clinical efficacy of influenza RdRp-targeting inhibitors has been primarily evaluated through the endpoint of time to symptom relief, a key measure reflecting the rapidity of clinical improvement following antiviral therapy (Supplementary Table 3) (Liu et al., 2021). Clinical trials have frequently reported that PA inhibitors are associated with rapid symptom relief and viral clearance.

#### 3.9.1 PA inhibitors

##### 3.9.1.1 BXM

In a dose-ranging phase 2 trial involving patients with uncomplicated influenza A(H1N1) pdm09, median time to alleviation of influenza symptoms (TTAS) was reported to be 23.4 to 28.2 h shorter in the BXM groups compared to the placebo group. Reductions in influenza virus titers were also reported to be significantly more rapid in all BXM treatment groups compared with placebo (Hayden et al., 2018). A Phase III study in patients with uncomplicated influenza reported that BXM (40 to 80 mg) shortened the median TTAS to approximately 53.7 h (95% CI, 49.5–58.5) compared with 77.0 h for oseltamivir and 85.6 h for placebo (Kawaguchi et al., 2018a). Early treatment (>12 to ≤24 h) further accelerated recovery and reduced viral titers in patients with ≤48 h from flu symptom onset (Kawaguchi et al., 2018a; Watanabe et al., 2019; Shah et al., 2020). Data from Phase II and III studies reported that ethnicity (Asian vs. non-Asian) did not significantly impact the drug effect on TTAS, with the time to symptom relief of 91.2 h in non-Asian patients and 62.4 h in Asians (Retout et al., 2022). Interestingly, a study reported that 8.4% of patients treated with BXM experienced biphasic fever, defined by an initial decrease in body temperature to below 37.5 °C for more than 24 h, followed by a subsequent rise to ≥37.5 °C (Saito et al., 2020).

Emerging PA endonuclease inhibitors, such as ZX-7101A, GP681, ADC189, TG-1000 exhibit comparable or improved efficacy outcomes in early-phase trials.

##### 3.9.1.2 ZX-7101A

ZX-7101A, administered as a single 40 or 80 mg dose, shortened the median time to symptom relief compared with placebo. In a Phase 2 trial, the median time to symptom relief was 34.7 h with 40 mg and 45.8 h with 80 mg, versus 63.6 h with placebo; in a Phase 3 trial, the corresponding values were 48.4 h and 39.4 h, respectively. ZX-7101A also accelerated viral clearance, with clearance occurring at approximately 43 h compared with 90.73 h in the placebo group. Notably, only 24.1% of patients initiated therapy within 24 h of symptom onset, a factor known to optimize antiviral efficacy. Therefore, the observed clinical and virological benefits may support the therapeutic potential of ZX-7101A even in a later-intervention cohort (Wang H. et al., 2025).

##### 3.9.1.3 GP681

GP681 has demonstrated significant reductions in time to symptom relief and viral RNA negativity, though variability in fever resolution suggests population-specific pharmacokinetic influences (Wang et al., 2025b). In the phase 2 trial, GP681 reported a median TTAS of 46.1 h with 20 mg to 50.0 h with 40 mg, and a median time to remission of fever of 27.0 h (40 mg) and 22.9 h with 20 mg (Wang et al., 2025b). However, in a phase 3 trial, though the median TTAS in GP681 treated patients was 42 h compared to 63 in placebo-treated patients, the median TTAS was not significant in females (*p* = 0.484) (Wang et al., 2025b). Moreover, a discrepancy was identified between the reported fever-reduction data and the corresponding Phase III graph. The graph shows a fever-reduction time of 16.65 h for placebo versus 13.52 h for GP681, which does not align with the values provided in the fever dataset (28.3 h for placebo versus 19.7 h for GP681). This inconsistency should be considered when interpreting the fever-resolution outcomes (Wang et al., 2025b).

##### 3.9.1.4 ADC189

In phase 2 and 3 clinical trials involving outpatients with uncomplicated influenza, ADC189 demonstrated significant antiviral efficacy with a median TTAS of 50.0 h compared with 68.1 h in patients treated with placebo (*p* < 0.0001) (Zhao et al., 2025). Furthermore, ADC189 (15 and 45 mg) shortened the median time to viral RNA clearance (50.7 and 45.8 h, respectively) compared with placebo (73.4 h), with a greater viral load reduction within 24 h of treatment initiation versus placebo (−2.32 log₁₀ vs. − 1.05 log₁₀ copies/mL) (Zhao et al., 2025).

##### 3.9.1.5 TG-1000

TG-1000 also demonstrated favorable symptom resolution consistent with other PA inhibitors, though detailed viral kinetics are yet to be fully characterized (Xu et al., 2023). A recent phase 3 trial in patients with acute uncomplicated influenza has reported a median alleviation time of 60.9 h with TG-1000 vs. 87.9 h with placebo. Furthermore, TG-1000 demonstrated significantly faster viral elimination rate compared with the placebo (91.6 h *vs.* 95.9 h) (Jin et al., 2024).

#### 3.9.2 PB1 inhibitors

##### 3.9.2.1 Favipiravir

Favipiravir has reported a median time to symptom relief ranging from 77.8–100.4 h in Phase II and Phase III trials (Hayden et al., 2022, 2024). Upon factoring in the time from onset to first dose, in patients with treatment onset ≥24 h, the median time to illness alleviation ranged from 76.0 to 79.0 h (Hayden et al., 2022). However, the efficacy of oral favipiravir in severe influenza remains insufficiently characterized. A retrospective study in critically ill adult patients compared favipiravir plus oseltamivir (23.8%) and oseltamivir monotherapy (76.2%). Combination therapy was reported to be associated with greater clinical improvement on day 14 (62.5% vs. 42.2%) and a higher proportion of patients with undetectable viral RNA on day 10 (67.5% vs. 21.9%) (Wang et al., 2020a).

#### 3.9.3 PB2 inhibitors

##### 3.9.3.1 ZSP1273

ZSP1273 has achieved intermediate outcomes, with a reported median TTAS of 46.9 h (200 mg BID), 54.9 h (400 mg BID), and 40.1 h (600 mg once daily [OD]) in a dose-ranging phase 2 trial in adults with acute uncomplicated influenza A infection (Yang et al., 2024). In subsequent Phase III trial, the median TTAS was shortened by 24.5 h with ZSP1273 (38.8 h) compared to placebo (63.4 h), and was similar to that of oseltamivir (42.2 h) (Yang et al., 2025).

##### 3.9.3.2 Pimodivir

Pimodivir showed early promise but was discontinued due to suboptimal efficacy and safety. (Trevejo et al., 2018; Finberg et al., 2019; Leopold et al., 2025).

The prevailing trend highlights the rapid onset of action and simplified dosing of PA inhibitors, whereas PB1 and PB2 inhibitors remain constrained by slower response and regimen complexity. Evidence gaps include head-to-head comparisons across inhibitor classes, evaluation of efficacy in high-risk populations, and real-world effectiveness studies. Collectively, these findings highlight the need for integrated strategies, such as combination therapy and optimized dosing windows to maximize clinical benefit and minimize resistance risk.

### 3.10 Safety

Clinical safety data have reported that RdRp-targeting inhibitors are generally well tolerated, with most adverse events (AEs) being mild to moderate and rarely leading to treatment discontinuation.

Among PA inhibitors, BXM is reported to be well-tolerated, with mild gastrointestinal and neuropsychiatric adverse reactions being occasionally documented (Tu et al., 2025). Common AEs include diarrhea in 25–46% of patients, and nausea/vomiting in 7–36% patients (Yoshino et al., 2020). In the FLAGSTONE study of patients with severe influenza, 2% patients reported AEs with fatal outcome, with 1–3% patients experiencing AE leading to treatment discontinuation (Kumar et al., 2022).

ADC189 has also reported an acceptable safety profile with any AE being reported in 35% patients, and nausea and diarrhea in 3–4% of patients (Wei et al., 2025; Zhao et al., 2025). Furthermore, studies have reported GP681 to be associated with AEs in 41–47% adults with acute uncomplicated influenza, with 31–36% being grade 1 or 2 (Han et al., 2024; Wang et al., 2025a, 2025b). Diarrhea (6.1%) and transient electrocardiographic abnormalities (sinus arrhythmia [4.8%] and sinus bradycardia [2.5%]) were also noted.

On the other hand, TG-1000 in healthy volunteers demonstrated manageable safety, with overall AEs ranging from 25 to 50%. Most events were mild laboratory or gastrointestinal disturbances without clinically significant hepatic or cardiac findings (Xu et al., 2023). Furthermore, ZX-7101A showed a favorable safety profile across Phase 1 to 3 studies (Wang H. et al., 2025; Wu et al., 2025b). In dose-escalation studies, it was well tolerated at doses up to 320 mg, with only mild gastrointestinal adverse events reported (Wu et al., 2025b). Low rates (7.1 to 9.2%) of mostly grade 1–2 treatment-related adverse events in Phase 2/3 trials were reported. Laboratory abnormalities were infrequent (≤4.6% neutrophil or WBC count decrease; ≤0.8% alanine aminotransferase/aspartate aminotransferase elevations), and no treatment discontinuations were reported (Wang H. et al., 2025).

Among PB1 inhibitors, favipiravir was associated with treatment-related adverse events in 25–28% adults with uncomplicated influenza with the most common being headache, diarrhea, and nausea (Hayden et al., 2022). However, pharmacovigilance data indicate a higher proportion of serious adverse drug reactions involving hepatic, renal, and cardiovascular systems, warranting careful monitoring (Negru et al., 2025).

For PB2 inhibitors, clinical data of ZSP1273 have consistently noted a higher incidence of gastrointestinal AEs, particularly diarrhea, which in some cases has been relatively severe. This class-specific adverse effect pattern underscores the need for careful monitoring in clinical applications of PB2-targeted antivirals (Ormond et al., 2017; Hu et al., 2021; Li C. et al., 2025; Pang et al., 2025).

Collectively, the prevailing trend is that RdRp-targeting inhibitors maintain acceptable safety profiles, with differences in AE patterns across classes. Evidence gaps include long-term safety data and systematic evaluation in high-risk populations. These findings emphasize the importance of continued surveillance and tailored risk–benefit assessments to ensure safe clinical integration of next-generation influenza antivirals (Supplementary Table 4).

### 3.11 Drug–drug interactions

Evidence indicates that metabolic characteristics and interaction potential vary substantially across RdRp-targeting inhibitor classes. Understanding these pathways is critical for predicting drug–drug interactions and optimizing dosing strategies, particularly in polypharmacy settings.

Several PA inhibitors, including BXM and ZX-7101A, show limited dependence on CYP-mediated metabolism, whereas GP681 represents an important exception because it undergoes substantial CYP3A4-mediated oxidative metabolism (Wang H. et al., 2025; Wu et al., 2025b). Clinical studies confirm that co-administration of BXM with NAIs such as oseltamivir does not result in clinically meaningful drug–drug interaction in healthy individuals (Kawaguchi et al., 2018b). The product label for ZX-7101A notes that no dose adjustment is required for mild to moderate hepatic impairment. Pharmacokinetic assessments also report no clinically significant drug–drug interactions of ZX-7101A with oseltamivir or other antivirals. A recent PK interaction study in healthy Chinese subjects reported that co-administration of ZX-7101A with itraconazole did not affect the T_max_ of ZX-7101. However, C_max_ was increased by 29.5% and the t_1/2_ was prolonged by 17.6%. Concomitant administration of multiple doses of itraconazole further increased ZX-7101 exposure after a single dose of 40 mg ZX-7101A; however, the observed exposure remained lower than that achieved with an 80 mg single dose of ZX-7101A alone. According to the product label, no dose adjustment is required when ZX-7101A is coadministered with itraconazole (Wu et al., 2025a).

In contrast to ZX-7101A, which does not require dose adjustment when co-administered with itraconazole, (Wu et al., 2025a) GP681 shows substantially greater metabolic dependence on CYP3A4. GP681 undergoes extensive oxidative metabolism, primarily via CYP450 3A4, with minor contributions from CYP1A2 and CYP2C19 (Han et al., 2024; Jiangxi Qingfeng Pharmaceutical Co. Ltd, 2024). Consequently, coadministration with drugs that inhibit or induce these enzymes, such as certain antibacterial or antiepileptic agents, may lead to altered systemic exposure, resulting in subtherapeutic efficacy or increased toxicity (Han et al., 2024; Jiangxi Qingfeng Pharmaceutical Co. Ltd, 2024). Controlled interaction studies show that co-administration with itraconazole (a potent CYP3A4 inhibitor) increases GP681 exposure (AUC by ~2.5-fold, C_max_ by 1.4-fold). Although these changes were not clinically significant and did not warrant dose adjustment in the published study, the product label recommends reducing the GP681 dose by half when co-administered with potent or moderate CYP3A4 inhibitors, indicating a precautionary approach based on regulatory assessment rather than peer-reviewed clinical evidence. Pharmacogenetic analyses confirm CYP3A4 as the primary metabolic pathway, highlighting the need for caution when GP681 is combined with strong CYP3A4 modulators (Han et al., 2024). Finally, the GP681 package insert now notes that the product should be used with caution in patients with moderate hepatic impairment (Jiangxi Qingfeng Pharmaceutical Co. Ltd, 2024).

PB2 inhibitors such as ZSP1273 have shown to maintain pharmacokinetic stability when combined with standard antivirals, with crossover studies confirming bioequivalence in key parameters despite minor fluctuations in C_max_ and AUC (Pang et al., 2025).

Collectively, these findings indicate that most RdRp-targeting inhibitors demonstrate favorable metabolic stability and low interaction potential, while enzyme-specific pathways in select agents require consideration in polypharmacy settings. However, current data are largely derived from Phase 1 studies in healthy volunteers, with limited evidence in high-risk populations or polypharmacy contexts. Comparative evaluations of metabolic variability across PA inhibitors and systematic interaction studies for PB1 and PB2 inhibitors remain scarce. Future research should prioritize pharmacogenetic profiling and real-world drug interaction assessments to guide safe clinical integration.

### 3.12 Efficacy and safety in high-risk populations

Current evidence on RdRp-targeting inhibitors in high-risk populations, including the elderly, immunocompromised individuals, and those with chronic comorbidities, is limited and uneven across drug classes.

Among PA inhibitors, BXM is the only agent with most substantial data available from clinical trials and post-marketing surveillance studies. The CAPSTONE-2 trial evaluated BXM in high-risk adolescents and adults, and reported reduction in influenza symptom duration (median TTIIS: 73.2 h) compared with placebo and oseltamivir (Ison et al., 2020). BXM also reported efficacy in patients with cardiovascular disease (median TTIIS: 69.8 h) (Collins et al., 2022) and chronic lung disease (median TTIIS: 77 h) (Cagas et al., 2022). However, comparable phase 3 data in high-risk populations remain unavailable for most next-generation PA inhibitors. In addition, several subsequent trials are ongoing to evaluate GP681 in high-risk patients and children with uncomplicated influenza (ClinicalTrials.gov, 2024; ClinicalTrials.gov, 2025c), and ZX-7101A in adolescents and children with influenza (ClinicalTrials.gov, 2025a, 2025b). For GP681, available subgroup data from the phase 3 trial in adults, adolescents and children did not show a statistically significant difference for fever or chills resolution in the children and adolescents subgroup; therefore, evidence supporting efficacy specifically in younger populations remains limited (Wang et al., 2025b).

Among PB1 inhibitors, evidence is sparse. Favipiravir (1,600 mg BID on day 1 and 600 mg BID on the following days or 1800 mg/800 mg BID) has been studied in critically ill adult hospitalized patients with influenza A or B and respiratory failure; however, target plasma concentrations were not consistently achieved, and robust efficacy data in high-risk cohorts are lacking (Wang et al., 2020b).

PB2 inhibitors such as ZSP1273 have limited data in special populations. A phase 1 study in participants with severe renal impairment indicated comparable PK to healthy controls and no need for dose adjustment, but broader evidence in elderly or immunocompromised patients is absent (Li H. et al., 2025).

Overall, beyond BXM no PA inhibitors have completed phase 3 trials in high-risk populations. PB1 and PB2 inhibitors lack systematic evaluation in elderly, immuno-compromised, or hepatically impaired cohorts. These findings highlight the need for targeted research prioritizing adequately powered studies in these groups to inform dosing strategies and safety profiles.

## 4 Discussion

### 4.1 Summary of evidence

This scoping review mapped the current evidence on influenza RdRp-targeting inhibitors across mechanistic, preclinical, pharmacokinetic, clinical, safety, resistance, drug–drug interaction, and high-risk population domains. Overall, PA inhibitors represent the most extensively studied and clinically advanced class, with BXM serving as the principal benchmark, and several next-generation PA inhibitors demonstrating similarly favorable early efficacy and pharmacokinetic profiles. In contrast, PB1 and PB2 inhibitors expand the mechanistic diversity of the antiviral pipeline but remain less mature in terms of comparative clinical evidence, resistance characterization, and dosing optimization. Across the included literature, the most consistent translational signal was observed for the PA inhibitors, which generally combines rapid antiviral activity with prolonged systemic exposure and simplified dosing. Clinical studies consistently reported a shortened time to symptom relief and accelerated viral RNA clearance with single-dose PA inhibitors compared with placebo or standard neuraminidase inhibitor-based therapies. By comparison, PB1- and PB2-targeted agents often showed less favorable PK durability, greater regimen complexity, or more limited evidence in late-phase clinical development.

The evidence base also highlighted antiviral resistance as a major cross-cutting challenge. Among PA inhibitors, amino acid substitution at the I38 residue emerged as the best-characterized mechanism of reduced drug susceptibility. Conversely, resistance mechanisms for PB1 and PB2 inhibitors remain comparatively less developed and are less consistently linked to definitive clinical outcomes. Although low PA-I38T emergence rates have been reported for both GP681 and ZX-7101A, their interpretation differs because ZX-7101A was analyzed using a clearly defined paired sequencing-evaluable population, whereas the GP681 denominator definition appears less transparent. Thus, the low numerical rates reported for GP681 should be considered encouraging but should not be interpreted as definitive evidence of lower resistance selection pressure than BXM or ZX-7101A. Standardized paired baseline/post-baseline sequencing approaches are needed to support reliable cross-compound comparisons.

Finally, for nearly all evaluated drugs other than BXM, robust clinical evidence in high-risk populations, complex drug–drug interaction settings, and real-world clinical practice remains limited, highlighting key gaps that warrant further clinical investigation.

### 4.2 Limitations

This review has several limitations. First, the evidence base remained heterogeneous with respect to study design, developmental stage, endpoints, and reporting quality, which limited direct cross-study comparisons. Second, because this was a scoping review, the objective was to map the available evidence rather than to perform a formal quantitative synthesis or comparative risk-of-bias-based effect estimation. Third, the available literature was weighted toward PA inhibitors, which may have amplified the maturity of this class relative to PB1 and PB2 inhibitors. Fourth, data in high-risk populations, polypharmacy settings, and special populations such as immuno-compromised or hepatically impaired patients were limited for most compounds, restricting conclusions about broader clinical applicability. An additional limitation relates to potential geographic and publication-availability imbalance in the evidence base. Although inclusion of Chinese-language databases strengthened the comprehensiveness, it also increased representation of compounds whose development programs, early clinical studies, or regulatory pathways are concentrated in China. As a result, this may limit the direct generalizability of some findings across regions, regulatory environments, and clinical practice settings.

### 4.3 Future perspective

Future development of influenza polymerase-targeting antivirals should improve durability, comparability, and clinical applicability. Although PA inhibitors currently lead the field, next-generation development should prioritize resistance-resilient designs, improvement of binding interactions against emerging variants, and combination approaches that reduce selection pressure while preserving rapid antiviral activity. For PB1 and PB2 inhibitors, progress will depend on improving pharmacokinetic durability, simplifying dosing regimens, and generating stronger translational evidence beyond early-stage development. Comparative studies across inhibitor classes remain important to determine whether differences in structure, target engagement, and exposure translate into meaningful clinical advantages. Additional priorities include population PK/PD modeling, pharmacogenetic evaluation, and systematic investigation in high-risk populations, including elderly, immunocompromised, and comorbid patients. Real-world effectiveness studies and drug–drug interaction assessments in polypharmacy settings are required for broader clinical integration.

## 5 Conclusion

This scoping review provides a comprehensive synthesis of the current landscape of influenza polymerase inhibitors, noting that PA-targeted agents currently have the most concentrated clinical evidence and the most extensive clinical development. While PA inhibitors have been widely studied across virological, pharmacokinetic, and clinical endpoints, head-to-head data remain lacking. Moving forward, ongoing resistance surveillance and optimization of clinical dosing regimens are expected to contribute to sustaining efficacy and minimizing resistance development. These efforts may support the advancement of next-generation polymerase inhibitors to provide durable and safe antiviral protection across diverse patient populations.
