# Supplementary material for: A scoping review of influenza RdRp-targeting inhibitors: mechanisms, clinical translation, and emerging challenges
Source: Front Microbiol. 2026 Jun 26;17:1860682. doi: 10.3389/fmicb.2026.1860682 (PMC13350557; doi:10.3389/fmicb.2026.1860682)
Supplement: Supplementary file 1 [file Data_Sheet_1.DOCX]

**Literature search strategy**

Pubmed and Embase search string

((influenza) AND (vRNP OR "viral ribonucleoprotein" OR "PB1 subunit" OR "PB2 subunit" OR "PA subunit" OR inhibitor OR antiviral) AND (clinical OR preclinical OR mechanism OR resistance) AND ("Sebaloxavir Marboxil" OR "ZX-7101A" OR Favipiravir OR "T-705" OR favipira OR favilavir OR Abigan OR Avifavir OR Areplivir OR J05AX27 OR Onradivir OR ZSP1273 OR "ZSP-1273" OR "Baloxavir marboxil" OR Xofluza OR J05AX25 OR "BXM (S-033188)" OR "S-033188" OR "S-033447" OR "BXA (S-033447)" OR ADC189 OR "ADC-189" OR "Deunoxavir Marboxil" OR TG1000 OR "TG-1000" OR "Pixavir Marboxil" OR "suraxavir marboxil" OR GP681 OR Mabaloxavir OR Masulaxavir OR Maseloxavir))

**SUPPLEMENTARY TABLE 1 Summary on the Preclinical Findings of RdRp-targeting inhibitors**

| **Inhibitor** | **Preclinical study findings** | **Viral titer** | **Model used** | **Key message** | **Reference** |
| --- | --- | --- | --- | --- | --- |
| BXM | IC_50_:  WT: 7.6 (1.7) nM  PA-I38T: 95.94 (5.24) nM  PA-I38M: 42.11 (17.52) nM  E23K: 19.65 (0.68) nM | Viral titers :7.95 (0.5), 7.45 (0.25) and 8.11 (0.28) logTCID_50_/mL for the WT, PA-I38T and PA-I38M mutants, respectively.  The viral titer of the E23K mutant at 72 hpi was 6.28 (0.28) logTCID_50_ (P < 0.05 vs the WT) | ST6GalI-MDCK cells using plaque reduction assay; Female BALB/c mice | E23K variant was associated with lower viral titers (up to 2 logTCID_50_/mL reduction) compared to the other recombinant viruses. | (Abed et al., 2020)^a^ |
|  | Cmax  Chickens: 171.3 ng/mL at 8 hpa → 0.14 ng/mL at 120 hpa; Ducks: 122.8 ng/mL at 8 hpa → 0.4 ng/mL at 120 hpa  AUC  Chickens: 8808.9 → 80.4 ng·h/mL (8–48 hpa) Ducks: 27,809.7 → 390.8 ng·h/mL (0.5–8 hpa) | 2.7 to 4.2 log_10_EID_50_/mL in chickens treated with 0.1 mg/kg and 0.5 mg/kg | Chickens: 20 mg/kg BID × 5 days;  Ducks: 20 mg/kg BID × 5 days | Single administration of 2.5 mg/kg of BXM was determined as the minimum dose required to fully protect chickens from HPAI virus. | (Twabela et al., 2020) |
|  | - | At 24 hpi, BXA (0.9 nM) inhibited rgH1N1-WT and rgH3N2-WT by 99.95 ± 0.02% and 99.2 ± 0.08%.  BXA reduced the titer of the PA-mutants rgH1N1-PA- PA-I38T and rgH3N2- PA-I38T only by 63 ± 3.23% and 60 ± 2.70%. | A549 cells | PA-I38T mutations were associated with reduced BXA susceptibility. | (Hamza et al., 2021) ^a^ |
|  | EC_50_ of BXA against various viruses ranged from 1.31 ± 0.35 to 153.82 ± 15.90 with a 1- and 116- fold change vs control, respectively. | Virus titer ranged from 185.0 ± 9.5 to 382.2 ± 4.3. | MDCK cells for A(H1N1)pdm09 viruses and MDCK-SIAT1 cells for A(H3N2) viruses | Substitutions I38L/M/S/T not only had a differential effect on BXA susceptibility (9- to 116-fold) but also on in vitro replicative fitness. | (Chesnokov et al., 2020) ^a^ |
|  | Median IC_50_:  A(H1N1)pdm09: 0.28 nM;  A(H3N2): 0.16 nM;  B/Victoria: 3.42 nM;  B/Yamagata: 2.43 nM | - | MDCK cells | No circulating A(H1N1)pdm09, A(H3N2), or B viruses had significantly reduced susceptibility to BXM and none of the viruses possessed an amino acid substitution at PA residue 38 during the 2017–2018 influenza season in Japan. | (Takashita et al., 2018) ^a^ |
|  | EC_50_:  A(H1N1)pdm09 WT: 50.12 (20.42) nM  PA-I38T: 4510.15 (1373.63) nM | - | MDCK cells; Mice | 67% of the population treated with BXM single therapy (1 or 5 mg/kg) possessed the treatment-emergent PA-I38X AAS variants (I38T, I38S, and I38V). | (Park et al., 2021) ^a^ |
|  | EC_50_:  0.12-0.48 (0.32) | 48 hpi, the EC_90_ of BXM was 1.2 (0.1) for a viral titer of 9.1 (0.1) log_10_TCID_50_/mL | Zoonotic and animal influenza A viruses in MDCK-SIAT1 cells | BXM showed broad-spectrum in vitro replication inhibi­tion of 4 types of influenza viruses (90% effective concen­tration range 1.2–98.3 nmol/L); susceptibility pattern was influenza A ˃ B ˃ C ˃ D. | (Mishin et al., 2019) ^a^ |
|  | IC_50_: 1–1.4 at 168 hpi to 3.5–8.2 at termination of treatment (day 28). | The virus titers in the lungs of the mice decreased from 7.1–7.5 log_10_ PFU/g at 7 days post infection to 4.7–5.9 log_10_ PFU/g, at day 28. | MDCK cells of nude mice infected with Influenza A virus | BXM increased the survival time of influenza virus-infected immunocompromised mice but did not result in virus clearance. | (Kiso et al., 2020b) |
|  | BXM (0.05, 0.5 or 5 mg/kg for A/PR/8/34; 0.5, 5 or 50 mg/kg for B/HK/5/72) orally bid for 1 day starting immediately after infection | At 72 h.p.i., significant decrease in virus titers of >2-log_10_ reduction compared to the vehicle control | MDCK cells  BALB/c mice | BXM reduced virus titers within 24 hours after initial treatment in a dose-dependent manner, with continued gradual decline thereafter. | (Fukao et al., 2019a) |
|  | Single dose BXA (8 mg/kg)  Cmax :21.1 ± 4.4 ng/mL at 24 h after administration | Upper respiratory tracts viral titer reductions of BXA vs placebo  1.7 (P = 0.03), 1.6 (P = 0.05), and 0.3 (P> 0.05) log_10_ 50% tissue culture infective dose(s) (TCID_50_)/mL at 48, 96, and 144 hpi, respectively.  AUC for viral shedding for BXA-vs placebo (16.3 ± 1.5 versus 23.3 ± 1.5; P = 0.007) | MDCK cells of ferrets infected with influenza B | BXM demonstrated antiviral potency against influenza B virus. | (Pascua et al., 2021) ^a^ |
|  | - | AUC of infectious viral load over the entire course of infection  Treatment with BXA, oseltamivir or placebo 24 hpi  BXA 7.07 (5.62)  untreated 21.10 (5.60)  oseltamivir 18.87 (5.17)  48 hpi  BXA 8.65 ± 1.70  placebo 13.06 ± 0.94 | MDCK cells of ferrets infected with influenza A(H1N1)pdm09 virus | BXA dosing was effective in reducing viral load in treated animals. Delayed antiviral treatment (from 24 to 48 hpi) was less effective in reducing viral transmission than early antiviral treatment. | (Lee et al., 2020) ^a^ |
|  | Median EC_50_ of the baseline viruses to BXA ranged from 1.3 to 1.6 nmol/L for A(H1N1)pdm09, from 0.74 to 1.4 nmol/L for A/H3N2, and from 5.6 to 8.5 nmol/L for type B virus, in the BXM 10, 20 and 40 mg and placebo groups.  3.59 and 0.73-fold change in ED50 for recombinant rgA/WSN/33 (H1N1) viruses with A36V and V545T mutations | At 24 hpi  Viral titers (logTCID_50_/mL) of  rgA/WSN/33  I38T −2.78  I38F −2.28  I38M −1.67 were lower than that of the WT, and rgA/Victoria/3/75 (H3N2) virus.  rgB/Maryland/1/59 with  I38F were lower than that of the  wild-type (−0.73 logTCID_50_/mL) | Canine kidney MDCK cells | Viruses harboring the I38T substitution showed impaired replicative fitness in cells. | (Omoto et al., 2018) ^a^ |
|  | - | Virus titer in the nasal swab  24hpi: 1 to 2.50 log_10_TCID_50_/mL  48hpi: 1.5 to 2.0 log_10_TCID_50_/mL  Post 48 hpi, virus titers were below the detection limit (0.67 log_10_TCID_50_/mL) | Female cynomolgus macaques infected with Dk/HE29-22  MDCK cells | In macaques, BXM treatment was associated with lower viral titers relative to comparator groups. | (Suzuki et al., 2021) ^a^ |
|  | Mean BXM EC_50_ of viruses  Pre-treatment samples (WT)  A(H1N1)pdm09: 1.1–1.5 ng/mL  A/H3N2: 0.31–0.39 ng/mL  Post-treatment viruses (PA/I38T)  82–87 ng/mL  36–49 ng/mL | At 48hpi, with 100x EC_50_ BXM, virus titer: 2.9 and 1.9 log_10_TCID_50_/mL lower for rgA/WSN/33 (H1N1) and rgA/Victoria/3/75 (H3N2) viruses, respectively, compared with the condition in the absence of BXM | Canine kidney MDCK and MDCK-SIAT1 cells  Ferrets infected with A/H1N1 and A/H3N2 | Although A/H1N1 and A/H3N2 viruses with reduced BXM susceptibility were able to replicate and transmit among ferrets, they had a moderate reduction in fitness compared to normal WT viruses, suggesting a reduced likelihood of spread. | (Lee et al., 2021) ^a^ |
|  | IC_50_ (nM) B/Quebec/MCV-11/2019: 53.2± 7  B/Quebec/MCV-11/2019-I38T: 731.3 ±239  rg-B/Washington/02/2019: 31.6 ± 1.9  rg-B/Washington/02/2019-I38T: 676 ± 144.2 | Viral titers for both MCV19 WT and I38T viruses reached a peak at 48 h p.i. with mean viral titers of 7.8 and 7.2 log TCID_50_/mL, respectively. A significant difference in viral growth titers between the two viruses was only observed at 24 h p.i. where MCV19 WT and its PA-I38T variant grew at mean titers of 5.49 and 3.95 log TCID_50_/mL, respectively (p < 0.05). | MDCK, ST6GalI-MDCK, 293T, and human airway epithelium | The I38T substitution increased the BXA IC_50_ value by 13.7-fold and resulted in reduced viral titers compared to the WT at early time points in ST6GalI-MDCK and at all time-points in human epithelial cells. | (Saim-Mamoun et al., 2022) ^a^ |
|  | Cmax:  In mice, C24 increased dose-dependently, measuring 26.1 ± 1.3 ng/mL at 10 mg/kg, 47.4 ± 4.9 ng/mL at 20 mg/kg, and 70.8 ± 13.9 ng/mL at 30 mg/kg.  In hamsters, C24 also rose linearly, reaching 17.3 ± 3.3 ng/mL at 10 mg/kg and 45.7 ± 19.7 ng/mL at 30 mg/kg. | At 120 hpi, BXA (120 mg/kg/day) reduced virus titers by 1.88 log_10_ TCID_50_/mL compared with vehicle.  At 144 hpi, BXA resulted in a significantly large drop in virus titers to below the lower limit of quantification (1.5 log_10_ TCID_50_/mL) in mice infected with the rgH1N1.WT and rgH1N1.NA/H275Y, as well as almost to the LLOQ in mice infected with the A(H1N1)pdm09.WT | MDCK cells  Mice infected with rgH1N1.WT or rgH1N1.PA/I38T and hamsters | BXA demonstrated dose-dependent antiviral effects in mice infected with PA/I38T-substituted strains. | (Kuroda et al., 2023) ^a^ |
|  | C_max_: 5.05–284 ng/mL  T_max_: 0.5 –2 h  T_1/2,z_ : 2.24–3.14 h  AUC _0-1 (ng.h/mL)_: 22.7–1690  AUC_0–12 (ng.h/mL)_: 22.0–1580 | Mean virus titers at 48hpi:  6.03 (0.35) to 6.55 (0.22) log_10_ TCID_50_/mL  24 h post-dosing, BXM (15 mg/kg q12h) resulted in ≥100-fold and≥10-fold reductions in influenza A and B virus titers. | MDCK cells  Mice infected with influenza A(H1N1), A(H1N1)pdm09, A(H3N2) or type B virus | Dose-dependent virus titer reductions were observed in mice. | (Ando et al., 2021) ^a^ |
|  | BXM 10 mg/kg:  C_max_: 165 (179) ng/mL  AUC _0-24h_: 715 (758) ng·h/mL  T_max_: 1.13 (0.63) h | BXM (10 and 30 mg/kg) showed reduction in virus titer (<0.5 log_10_ TCID_50_/mL) at 48hpi. | MDCK cells  Ferrets | Single-day oral dose of BXM showed reduction in virus titers and symptoms on 1 day after administration in ferrets infected with influenza A virus. The suppression of body temperature changes over time from 8 hours up to 48hpi was also significantly greater with BXM than vehicle. | (Kitano et al., 2020) ^a^ |
|  | A(H1N1) pdm09  EC_50_ ranged from 0.6 ± 0.2 (WT) to 557.1 ± 44.6 (E23R + I38T)  A(H3N2)  EC_50_ ranged from 0.3 ± 0.1 (WT) to 975.0 ± 85.1 (E23R + I38T) | A(H1N1)pdm09 E23R or E23R + I38T took 72–96 hpi to reach WT-equivalent titers | MDCK cells | The E23R mutation may have the potential to impact BXM treatment efficacy. | (Jones et al., 2022) ^a^ |
|  | EC_50_:  WT: 8.46 ± 4.33  H1N1: *<*0.25  Coinfection (WT): *>*16.00  Coinfection (H1N1): *<*0.25 | - | Calu-3 cells | Combination of molnupiravir and BXM should be considered for early treatment in patients with SARS-CoV-2 and influenza coinfection | (Liu et al., 2024) ^a^ |
| Favipiravir | EC_50_:  PB1 WT  A/PR/8/34 (H1N1): 5.98 (38.1) μg/mL  A/Osaka/1480/96 (H3N2): 4.83(30.7) μg/mL  PB1 K229R  A/PR/8/34 (H1N1): 44.1 (281) μg/mL  A/Osaka/1480/96 (H3N2): 53.9 (343) μg/mL | - | Human embryonic kidney 293T cells | PB1 K229R mutant was resistant to favipiravir, suggesting that the amino acid at position 229 in PB1 of influenza virus may play a pivotal role in polymerase activity. | (Komeno et al., 2022) |
|  | IC_50_:  A(H1N1)pdm09, H3N2, H7N9, B: range 0.14–0.99 μM  EC_50_:  A(H1N1)pdm09, H3N2, H7N9, B: range 2.92–38.72 μM | - | MDCK cells | None of the tested viruses had statistically significant reduced susceptibility to favipiravir. | (Takashita et al., 2016) |
|  | EC_50_:  K09: 8.39 (3.76–18.74) μM  rK09/NA:Y275: 14.66 (6.54–32.86) μM  K/2785: 10.82 (5.93–19.76) μM | Viral titers in lungs [log_10_(PFU/mL/g)] at 216 hpi  decreased from 7.42 (0.26) to 5.52 (0.07) upon combination of peramivir (100 mg/kg/day) with favipiravir (20 mg/kg/day) | MDCK cells; female DBA/2 mice | Increasing favipiravir concentrations were associated with improved survival and reduced viral replication in lungs in animal models, in a favipiravir concentration-dependent manner. | (Park et al., 2014) |
|  | - | Virus titers were reduced significantly at 96 hpi by combination therapy containing favipiravir combined with peramivir at 0.25 and 0.5 mg/kg/d. | Female BALB/c mice | Monotherapy with favipiravir provided 40, 70, and 100% protection against lethal infection following administration of 20, 40, and 100 mg/kg/d, respectively | (Tarbet et al., 2012) |
|  | IC_50_:  SH5190 virus: 3.10 (1.67–5.76) μM  SH5190 R292K mutant: 6.26 (4.96-7.90) μM | - | MDCK cells | Favipiravir inhibited both the variant and the WT. | (Zhang et al., 2014) |
|  | IC_50_:  Influenza A(H1N1)pdm  WT: 31.07 (7.86); 57.96 (8.90) nM  PA-I38T: 35.07 (6.34); 40.65 (15.85) nM  Influenza A(H3N2):  WT: 136.20 (41.54); 30.38 (7.83) nM  PA-I38T: 73.87 (9.86); 59.65 (7.27) nM | - | MDCK cells, ST cells | All viruses tested were susceptible to favipiravir. | (Takashita et al., 2023) |
|  | IC_50_:  <1 μg/mL against WT, E119D, and E119D/H275Y recombinant A(H1N1)pdm09 virus | - | MDCK cells transfected with the cDNA of human 2,6-sialyltransferase | The mutant viruses remained susceptible to favipiravir | (L’Huillier et al., 2015) |
|  | EC_50_ of 50 mg/kg/day favipiravir:  B/Memphis/20/96 R152: 4.50 (1.06) μM  B/Memphis/20/96 R152K: 7.49 (1.67) μM | T-705 administration also significantly reduced viral loads and suppressed pulmonary pathology | C57BL/6 mice infected with wild-type or oseltamivir-resistant influenza B/Memphis/20/96 viruses | Favipiravir protected mice from lethal infection in a dose-dependent manner without emergence of favipiravir -resistant viruses after favipiravir treatment. | (Fang et al., 2020) |
|  | EC_50_:  H1N1: 2.0 (0.5)  H3N2: 1.4 (0.8)  H5N1: 4.3 (1.9) | - | MDCK cells; Mice | Favipiravir alone inhibited viruses in cell culture at 1.4 to 4.3 μM. T-705 was ≥70% protective at 50 to 100 mg/kg/day but inactive at 25 mg/kg/day. | (Smee et al., 2010) |
|  | IC_50_: 2.7 μM  EC_50_: 11 μM | Viral RNA synthesis is completely inhibited by favipiravir ≥50 uM | MDCK cells | Favipiravir acts as a potent and GTP-competitive inhibitor of the viral polymerase. | (Vanderlinden et al., 2016) |
|  | IC_50_: 3.01 (0.03)–6.61 (0.02) μM  EC_50_: 12.95 (0.73) –17.82 (0.27) μM | Mean infectious titers  decreased (from 6.7 log_10_ PFU/mL to undetectable; *P <* 0.0001) during passages 6 to 24 | MDCK cells and human lung carcinoma (A549) cells | Favipiravir was associated with mutagenic activity in influenza viruses. | (Baranovich et al., 2013) |
|  | IC_50_  WT: 3.6 (0.79–14)  PA-I38T: 1.7 (0.91–3.0)  NA-R152K: 2.2 (1.1–4.1)  PA-I38T/ NA-R152K: 1.8 (0.85–3.7) | Lung viral titers (log_10_ PFU ± SD/g) decreased from  7.2 (0.2) to 6.0 (0.1) in WT  7.4 (0.2) to 5.8 (0.8) in PA-I38T  7.7 (0.2) to 5.3 (0.4) in NA-R152K  7.2 (0.6) to 5.3 (0.1) in PA-I38T/ NA-R152K with 50 mg/kg FAV at 72 hpi | MDCK cells  Mice | Favipiravir treatment provided protection to mice from lethal infection with all viruses tested. | (Kiso et al., 2023) |
|  | Mean EC_50_: 2.6 to 12.1 μM | The mean virus titers ranged from 6.3 (day 1 after virus exposure) → <1.75 log₁₀ (day 7 after virus exposure)  The virus titer after 24 h of  300 mg/kg/day of favipiravir ranged from 2.4→3.5  100 mg/kg/day of favipiravir ranged from 4.9→5.3  30 mg/kg/day of favipiravir: 5.8 | MDCK cells  Influenza A (H5N1) virus infected mice | Favipiravir administered per os once, twice, or four times daily for 5 days beginning 1 h after virus exposure was highly inhibitory to the influenza A infection. | (Sidwell et al., 2007) |
|  | IC_50_ (µg/mL)  GD/3: 0.47  rGD/3-NA294R: 0.67  rGD/3-NA294K: 0.86  Anhui/1: 0.35 | At 144 hpi, the mean virus titers of all three HPAI H7N9 viruses in the ferret lungs (mean titers = 5.3, 4.8, and 4.4 log_10_ [PFU/g], respectively) were higher than the mean virus titer of the LPAI Anhui/1 virus (mean titer = 1.9 log_10_ [PFU/g]),  Statistically insignificant differences for rGD/3-NA294K virus | Ferrets infected with the three HPAI H7N9 viruses administered 60 or 150 mg/kg per 200 mL of favipiravir orally twice daily for 5 days after infection | Favipiravir was active against all viruses tested, suggesting that it may be an effective treatment option against NA inhibitor-resistant HPAI H7N9 viruses. | (Imai et al., 2017) |
|  | - | Virus Titer, Mean Log_10_ PFU ± SD/g  At day 14 after 5-day treatment  Favipiravir (20 mg): 7.3 (0.3)  Favipiravir (30 mg): 7.1 (0.5)  Laminvair+Favipiravir (20mg): 7.2 (0.3)  Laminvair+Favipiravir (30mg): 7.0 (0.1)  At day 14 after 28-day treatment  Favipiravir (20 mg): 6.2 (0.2)  Favipiravir (30 mg): 6.3 (0.1)  Laminvair+Favipiravir (20mg): 3.4 (0.4)  Laminvair+Favipiravir (30mg): 5.7 (0.5) | Nude (BALB/c-*nu/nu*) mice Infected with Influenza A Virus | Combination therapy of favipiravir and NAI in immunocompromised hosts was associated with increased survival duration. | (Kiso et al., 2018) |
|  | EC_50_ ranged from 1.9 to 7.8. | Virus titer decreased from 5.7 (0.3) to ≤0.5 with combination of 100 μM favipiravir and NAIs. | Influenza A (H1N1) virus isolates in MDCK cells. | Combination therapy with favipiravir and NAIs for treatment of human influenza virus infections was observed to be effective. | (Tarbet et al., 2014) |
|  | - | 6.9 to 2.0 72 hpi in A/California/04/09 (H1N1) at 168 hpi, the virus titer ranged from 5.1 to undetectable traces. | All swine-origin H1N1 viruses in MDCK cells  Mice | Favipiravir and NAIs may represent a treatment option against S-OIV pandemic strains. | (Itoh et al., 2009) |
|  | EC_50_s of 0.19 uM (0.03 ug/mL) to 5.03 uM (0.79 ug/mL) | Favipiravir completely inhibited plaque formation at concentrations of 15.9 uM (2.5 ug/mL) | MDCK cells | Favipiravir was reported to inhibit in vitro replication of a wide range of influenza viruses. | (Sleeman et al., 2010) |
|  | IC_50_s from 0.013 to 0.48 ug/mL against Influenza A, B and C. | 100 mg/kg of body weight/day (four times a day) for 5 days significantly reduced the mean pulmonary virus yields and the rate of mortality in mice infected with influenza virus A/PR/8/34 (3 X 10^2^ PFU) | MDCK cells  Mice | Favipiravir was reported to be highly selective against influenza virus infections | (Furuta et al., 2002) |
|  | - | Plaque formation of influenza A/PR/8/34 virus was completely inhibited by 10 μg/mL of favipiravir after 72 hpi.  In mice infected with a high challenge dose of influenza A/PR/8/34 virus, orally administered T-705 (200 and 400 mg/kg/day) completely prevented the death of mice | MDCK cells  BALB/c mice | Favipiravir demonstrated potent inhibition of influenza virus infections | (Takahashi et al., 2003) |
|  | T_1/2_: 5.6 ± 0.6 hours | 100 mM caused the greatest reduction in virus titer, even the 2 h incubation with T-705  10 mM treatments for 4, 6 and 8 h  were as inhibitory as the same concentration applied for 24 h. | MDCK cells | Short-term incubation of favipiravir with H5N1 virus-infected cells was predicted to provide an antiviral benefit. | (Smee et al., 2009) |
|  | T-705RTP, metabolized from T-705 (favipiravir) via a cellular phosphorylation pathway, may act as a pseudopurine or a pseudopurine nucleoside or nucleotide in human cells | 30 mg/kg/day favipiravir reduced lung viral titers from 5.8 (0.2) at day 3 to  4.4 (0.5) 1hpi  5.0 (0.1) 24 hpi  4.7 (1.0) 48 hpi  By 216 hpi, the viral titers were undetectable. | Mice | Favipiravir provided protection mice from lethal H5N1, including oseltamivir-resistant strains. | (Kiso et al., 2010) |
|  | IC_50_  Favipiravir: 1.6 to 2.3  Favipiravir+ Antistem mAb: 1.7 to 2.3  Favipiravir + Anti-RBS mAb: 1.1 to 4.7 | Mean viral titer  Favipiravir  168 hpi: 6.1±0.2  336 hpi: 5.9±0.3  672 hpi: 5.5±0.3  Favipiravir+ Antistem mAb+ Anti-RBS mAb  <1.7 at all timepoints | Nude mice infected with influenza A/MA-CA04 | Triple combination therapy of favipiravir plus anti-Stem and anti-RBS mAbs completely stopped virus replication in nude mice, resulting in virus clearance. | (Kiso et al., 2020a) |
|  | - | Significant decreases in lung virus titers were noted in groups treated with favipiravir alone (at 10, 30 or  100 mg/kg/day) or with favipiravir combined with oseltamivir (at 3 mg/kg/day). | Female BALB/c mice | Oseltamivir and favipiravir in combination were effective against H1N1 influenza virus | (Smee et al., 2013) |
| BXM and Favipiravir | EC_50_  WT A(H1N1) pdm09  Favipiravir: 4.05 (0.88) uM  A/California/7/2009  BXM: 0.48 (0.22) nM  A(H3N2) virus  Favipiravir: 10.32 (1.89) uM  A(H3N2) strain  BXA: 19.55 (5.66) nM | 48 hpi viral titers  A(H1N1)  Untreated: 4.4 (3.6) X 108 TCID50/mL  10 nMBXA: 5.6 (4.3) X 105 TCID50/mL.  A(H3N2)  Untreated: 7.5 (6.4) X 109  10 nM BXA: almost complete inhibition  5nM BXA at 72 hpi: 4.9 (1.78) X 108 | ST6-GalI-MDCK cells | BXA was effective by inducing a 3 log_10_ reduction in mean peak viral titers.  Combination therapy is more potent than monotherapy. | (Checkmahomed et al., 2020) |
|  | IC_50_ (nM) against dual H275Y mutant influenza A(H1N1)pdm09 viruses  BXM: 3.49 to 11.04  EC_50_ (uM)  Favipiravir: 4.76 to 15.13 | - | MDCK cells | All of the dual H275Y mutant viruses tested were susceptible to BXM and favipiravir | (Takashita et al., 2020) |
|  | IC_50_  WT A(H1N1)pdm09  Favipiravir: 31.07 ± 7.86 uM  BXM: 0.88 ± 0.12nM  PA I38T A(H1N1)pdm09  Favipiravir: 35.07 ± 6.34 uM  BXM: 98.19 ± 10.33 nM  Influenza B:  WT  Favipiravir: 23.29 ± 1.10  BXM: 46.79 ± 2.93  PA I38T  Favipiravir: 15.56 ±5.54  BXM: 680.97 ± 21.18  Influenza C:  Favipiravir: 13.20 ± 1.15 to 28.62±4.85  BXM: 6.20 ±0.92 to 24.83 ± 9.48  Influenza D:  Favipiravir: 12.48 ±2.71 to 35.83 ± 12.83  BXM: 44.51 ± 9.12 to 51.02±12.11 | - | MDCK and ST cells | All viruses tested were susceptible to both BXM and favipiravir | (Takashita et al., 2023) |
|  | EC90 (nM±SD)  BXA: 0.62 (0.11) to 1.13 (0.58)  Favipiravir: 12,014.40 (7,990.32) to 18,879.83 (13,666.58) | BXA 4nM achieved a 1.5–2.8 log reduction in virus titers of A(H7N9), including the NA-R292K mutant virus and highly pathogenic avian influenza viruses.  In mice, oral administration of BXM at 5 and 50 mg/kg twice a day for 5 days completely protected from a lethal A/Anhui/1/2013 (H7N9) challenge, and reduced virus titers more than 2–3 log in the lungs. | MDCK cells  Mice | BXM exhibits therapeutic effects against A/Anhui/1/2013 (H7N9) in mice when BXM treatment is delayed up to 48 hpi. | (Taniguchi et al., 2019) |
|  | - | - | - | Novel compound 54 showed antagonistic effect when combined with either favipiravir or BXM. | (Bonomini et al., 2024b) |
| 3′-Azido-3′-deoxy-thymidine-5′-triphosphate | IC_50_:  1.2 μM | - | Homology model of IAV RdRp | AZT-TP binds to the PB1 catalytic site near to the tip of the priming loop. | (Pagadala, 2019) |
| Deunoxavir marboxil (ADC189) | EC_50_ (nmol/L)  A/WSN/33-PA/I38T: 1.73  A/WSN/33: 2.52 | At 72 hpi, viral titers in mice treated with 0.2 mpk, 1 mpk, and 10 mpk doses of ADC189 decreased by 1.88 log_10_(plaque numbers/g lung), 2.19 log_10_ (plaque numbers/g lung), and 2.64 log_10_(plaque numbers/g lung), respectively | BALB/c mice | ADC189 demonstrated antiviral efficacy in the study**.** | (Wei et al., 2025) |
| ATV2301  15 mg/kg (PO) in CD-1 mice  1 mg/kg (IV) in CD-1 mice | EC_50_:  H1N1 = 1.88 nM,  H3N2 = 4.77 nM  Safety index: H1N1 = 18218  C_max_: 4,073 (1,134); T_max_: 0.42 (0.14)  T_1/2_: 4.30 (0.77); AUC _(0–∞)_ (ng.h/mL): 11,085 (1,238)  C_max_: 940 (34.5); T_max_: 0.08 (0.00)  T_1/2_: 3.43 (0.01); AUC _(0–∞)_ (ng.h/mL): 1,228 (109) | Lung index and virus titer indicated that the antiviral effect of ATV2301A was comparable to BXM | MDCK cells  BALB/c mice models | ATV2301 exhibited anti-influenza activity and high stability in both human and mouse liver microsomes, with comparable lung viral titers to that of BXM. | (Chen et al., 2024a) |
| ATV03, ATV07  ATV03  15 mg/kg (PO) in CD-1 mice  1 mg/kg (IV) in CD-1 mice  ATV07  1 mg/kg (IV) in CD-1 mice  1 mg/kg (IV) in CD-1 mice | EC_50_:  Influenza A H3N2  ATV03: 0.78 (0.10) nM; SI>64103  ATV07: 0.78 (0.01) nM; SI=31603  Influenza B  ATV03: 2.02 (0.40) nM  ATV07: 2.32 (0.29) nM  C_max_: 548 (109); T_max_: 1.33 (0.58)  T_1/2_: 2.83 (0.08); AUC _(0–∞)_ (ng.h/mL): 2,790 (770)  C_max_: 211 (43.70); T_max_: 0.08 (0)  T_1/2_: 2.6 (1.47); AUC _(0–∞)_ (ng.h/mL): 343 (67.60)  C_max_: 222 (87.20); T_max_: 2.67 (1.15)  T_1/2_: 4.45 (0.84); AUC _(0–∞)_ (ng.h/mL): 1678 (488)  C_max_: 258 (28); T_max_: 0.08 (0)  T_1/2_: 1.99 (0.15); AUC _(0–∞)_ (ng.h/mL): 355 (22.30) | - | MDCK cells and CD-1 mice | Preclinical assays suggested inhibition of PA-associated endonuclease/RdRp activity and effects on NP-related viral replication markers. | (Chen et al., 2024b) |
| Onradivir (ZSP1273) | t_1/2_: 2.11 ± 1.46 h  AUC_0-last_: 3950 ± 773 ng·h/mL  IC_50_:  0.014 (0.006) – 0.777 (0.427) nM  EC_50_:  0.012 (0.002)–0.063 (0.028) nM against multiple influenza strains | At 120 hpi, ZSP1273 (3, 10, and 30 mg/kg) significantly reduced lung viral titers in a dose-dependent manner, 2.37 log_10_, 4.18 log_10_, and 4.97 log_10_, respectively.  At 192 hpi, the lung viral titers were all 0. | MDCK cells  SD rats | ZSP1273 was associated with a dose dependent decrease in viral load against multidrug-resistant strains. | (Chen et al., 2023) |
| Sebaloxavir marboxil (ZX-7101A) | IC_50_: 21.72 nmol/L  EC_50_s against pH1N1, H3N2, H7N9 and H9N2 were 4.13 nmol/L, 1.03 nmol/L, 4.39 nmol/L, and 2.74 nmol/L, respectively^b^ | Treatment of ZX-7101A significantly decreased viral RNA loads in mouse lungs. | MDCK cells, HEK293T cells  BALB/c female mice | ZX-7101A was associated with a broad-spectrum antiviral potency against various IAV subtypes, including pH1N1, H3N2, H7N9 and H9N2 | (Luo et al., 2023) |
| Compound 3 (novel) | T_1/2_: 1.242 h  Clearance rate: 1.017 mL/min/kg  Plasma protein binding: 88.28% | - | Homology modelling (PDB: 6FS8) | Molecular modeling predicted that Compound 3 may retain binding against BXM-resistant variants, but experimental validation is required. | (Ren et al., 2024) |
| Novel 1,3-oxathiolane nucleoside derivatives of favipiravir | IC_50_: 40.4 umol/L | Viral suppression rate ranged from  33.3 to 50% (30 ug/mL)  75 to 100% (100 ug/mL) | MDCK cells | A class of 1,3-oxathiolane nucleoside derivatives of T-705 was designed and synthesized, and one of them was identified as a novel scaffold against viral infection. | (Han et al., 2018) |
| Novel cycloheptathiophene-3-carboxamide derivative* | EC_50_ value of 89 nM against IAV strain (A/PR/8/34 - H1N1) coupled with a CC_50_ value of 224 μM in MDCK cells, | At 24 hpi, EC_50_ of Compound 45 in Human A549 cells: 0.087±0.021 | MDCK cells | Compound 45 represented the most potent anti-IV compound acting by inhibiting PA-PB1 interaction | (Bonomini et al., 2024a) |
| Novel 2,3-dihydro-6,7-dihydroxy-1H-isoindol-1-one derivatives | Four compounds displayed EC_50_ ~4 μM | - | Molecular docking (PDB: 4E5H) | Compound 17 was shown to retain full activity against the BXM-resistant I38T mutant | (Rogolino et al., 2021) |

**Note:** BXM is the prodrug of BXA; in vitro antiviral activity assays generally evaluate BXA unless otherwise specified.

^a^These studies report the pharmacokinetic properties of BXA.

^b^The EC50 values are indicative of ZX-7101, the active form of ZX-7101A.

*Novel cycloheptathiophene-3-carboxamide derivative (Compound 45) is an exploratory background compound and was not included in the formal evidence synthesis.

AUC_₀–∞_: area under the plasma concentration–time curve from time zero to infinity; AUC₀–t: area under the plasma concentration–time curve from time zero to last measurable concentration; A/PR/8/34: Influenza A/Puerto Rico/8/34 strain; A549: Human type II respiratory epithelial cells; ATV03: Substituted dibenzoxepine-based baloxavir derivative 03; ATV07: Substituted dibenzoxepine-based baloxavir derivative 07; BALB/c mice: Inbred laboratory mouse strain; BXA: Baloxavir acid; BXM: Baloxavir marboxil; C_max_: maximum plasma concentration; E23K: Glutamic acid to lysine substitution at position 23; EC_50_: Half Maximal Effective Concentration; HEK293/293T: Human embryonic kidney 293/293T cells; H1N1: Influenza A H1N1 subtype; H3N2: Influenza A H3N2 subtype; H5N1: Influenza A H5N1 subtype; h, hours; hpi, hours post infection; HPAI, highly pathogenic avian influenza; IC_50_: 50% inhibitory concentration; MDCK: Madin-Darby canine kidney cells; NHBE: Normal human bronchial epithelial cells; NAIs: Neuraminidase inhibitors; PA: Polymerase acidic protein; PB1: Polymerase basic protein 1; PB2: Polymerase basic protein 2; PR8: A/Puerto Rico/8/34 strain; R152: Arginine at position 152; RTP/RMP: Ribonucleoside triphosphate / monophosphate; SI: Selectivity index; ST cells: Simian testicular cells; WT: Wild type.

**SUPPLEMENTARY TABLE 2 Summary of Human Pharmacokinetic Properties of Representative Influenza RdRp-targeting Inhibitors**

| **Inhibitor/Drug** | **Dose** | **C_max_ (ng/mL)** | **T_max_ (h)** | **T_1/2_ (h)** | **CL/F (L/h)** | **AUC _(0–∞)_**  **(ng·h/mL)** | | **AUC _(0–t)_ (ng·h/mL)** | | **Reference** |
| --- | --- | --- | --- | --- | --- | --- | --- | --- | --- | --- |
| BXM | 40 mg and 80 mg | - | - | - | - | Low: 0.72–5.13; High: 5.13–16.65 | | - | | (Retout et al., 2022) |
|  | 6–80 mg | 6.92 | - | 48.9–90.9 | - | - | | - | | (Kawasaki et al., 2016) |
|  | 10 mg, 20 mg, 40 mg | 14.8 → 57.1 | - | - | - | - | | - | | (Watanabe et al., 2019)^a^ |
|  | 6, 20, 40,60, 80 mg | 11.0 (22.3) → 253 (23.9) | 1.5 (1.0, 12.0) → 4.0 (1.0, 5.0) | 48.9 (30.1) → 114 (19.3) | - | 1018 (35.7) → 11970 (27.8) | | 417.4 (22.1) → 6795 (25.5) | | (Koshimichi et al., 2018)^a^ |
|  | 40 mg | 137 (42.7) | 4.00 (3.00–5.00) | - | - | 6,764 (30.5) | | 6,574 (29.5) | | (Kawaguchi et al., 2018b) |
|  | 20, 40, 80 mg | 41.4 (12.9)–206.2 (78.3) | 3.5 (range: 1.5-4) –4 (range: 3-5) | 80.8 (6.9)–98.3 (23.5) | 7.6 (2.3)-10.1 (6.1) | 2,864 (887)–9503 (2198) | | 1134 (295)-5393 (1776) | | (Kim et al., 2022) |
|  | 40 and 80 mg | 40 mg: 107.6 (24.2)  80 mg: 206.9 (38.3) | 40 mg: 4 (3-6)  80 mg: 4 (3-5) | 40 mg: 99.74 (18.0)  80 mg: 88.89 (17.1) | 40 mg: 4.866 (25.5)  80 mg: 7.019 (29.4) | 6,955 (25.5)–9,643 (29.4) | | 6,442 (24.3)–9,218 (29.2) | | (Liu et al., 2022)^a^ |
| Favipiravir | 1800 mg BID | Favipiravir (US316):47,500 Favipiravir (US317):42,100 | Favipiravir (US316):1 Favipiravir (US317):1 | - | - | - | | - | | (Hayden et al., 2022) |
|  | 1800 mg | 47.5 (27.2) | 1 | - | - | - | | - | | (Hayden et al., 2024) |
| Onradivir (ZSP1273) | 600 mg QD | 6421.7 (3660.6) | 0.88 (range: 0.25-4.01) | 17.8 (33.6) | 47.4 (17.8) | 18392.1 (12058.8) | | 16529.6 (7808.2) | | (Pang et al., 2025) |
|  | 100–1200 mg, Fasted, Fed, 200 mg QD/BID (F 1 & 5), 400 mg BID (Day 1 & 5), 600 mg BID (Day 1 & 5) | 747.67 (362.36) → 7952.50 (3490.39) | 0.50 (range: 1.5-0.5) → 2 (range: 3-1.5) | 12.08 (3.30) → 34.98 (42.42) | 33.34 (12.07)→55.77 (18.05) | 1958.44 (768.56) → 26407.92 (9270.96) | | 1879.33 (785.41) → 26182.65 (9252.49) | | (Hu et al., 2021) |
|  | 600 mg | Mild hepatic impairment: 11,700 (6060) Moderate hepatic impairment: 11,600 (7850) Normal hepatic function: 5420 (2060) | Mild: 1.25 (range: 0.50–2.00) Moderate: 1.50 (range: 0.50–3.01)  Normal hepatic function: 1.25  (range: 0.5-4.0) | Mild: 12.5 (4.65) Moderate: 19.9 (15.3)  Normal hepatic function: 11.9 (4.84) | Mild: 26.9 (9.65) Moderate: 16.4 (9.18)  Normal hepatic function: 43.6 (10.1) | Mild: 25,600 (11,100) Moderate: 50,300 (29,900)  Normal hepatic function: 14400 (3080) | | Mild: 25,400 (11,100) Moderate: 48,600 (29,300) Normal hepatic function: 14300 (3040) | | (Li et al., 2025a) |
|  | 600 mg in patients with renal impairment | 6,848.10 (77.78) | 1.48 (range- 0.75-2.95) | 12.62 (50.03) | 39.18 | 15,314.36 | | 15,071.73 | | (Li et al., 2025b) |
| Suraxavir Marboxil (GP681)^b^ | 20,40 mg | GP1707D07 C24 (24.9 ± 9.5 ng/mL and 16.0 ± 5.9 ng/mL). | - | - | - | | - | | - | (Wang et al., 2025b) |
|  | 20 mg | 19.3 (11.2) | 4.0 (range: 2.0–6.0) | 65.8 (12.1) | 21.1 (10.6) | | 1,122.9 (429.5) | | 1,040.1 (410.2) | (Han et al., 2024) |
| Deunoxavir marboxil (ADC189) | 15, 30, 45, 60, 75, 90 mg | 18.08 (11.79) → 181.89 (19.04) | 3.49 (range: 3-6) → 5.99 (range: 1-6.01) | 76.69 (23.90) → 98.28 (26.30) |  | | 1,253.76 (24.23) → 8,303.32 (26.42) | | 1,181.82 (20.15) → 7,892.87 (26.30) | (Wei et al., 2025) |
| Pimodivir | 100, 400, 600, 900, 1,200 mg | 131 (94.0) →1,640 (59.0) | 2 (range: 1-6) →4 (2-12) | - | - | | - | | 591 (66.9) →8,580 (42.6) | (Trevejo et al., 2018) |
| Sebaloxavir marboxil (ZX-7101A)^c^ | 40, 80, 160, 240, 320 mg | 109.6 (18.6) - 805.6 (175.0) | 3 (range: 3-4) –4 (range: 0.5-5) | 83 (14.8) –125.6 (45.9) | - | | 8,225 (1699.1)–37,042.2 (8691.1) | | 7,183.6 (1130.8)–35,671.8 (8311.1) | (Wu et al., 2025) |
| Pixavir marboxil (TG-1000)^d^ | 10, 20, 40, 80, 120, 160 mg | 22.2 (60.8)–446 (14.5) | 3.02 (range: 2-4) –6 (range: 4-6) | 33.8 (7.9)–39.4 (12.6) |  | | 857 (46.3)–10,100 (19.1) | | 1,180 (18.9) –10,300 (19.3) | (Xu et al., 2023) |

^a^These studies report the pharmacokinetic properties of BXA, the active metabolite of BXM.

^b^These pharmacokinetic properties are derived from the analysis of GP1707D07 (the active metabolite of GP681)

^c^These pharmacokinetic properties are derived from the analysis of ZX-7101, the active metabolite of ZX-7101A.

^d^These pharmacokinetic properties are derived from the analysis of TG-0527, the active metabolite of TG-1000

AUC_₀–∞_: area under the plasma concentration–time curve from time zero to infinity; AUC₀–t: area under the plasma concentration–time curve from time zero to last measurable concentration; BXA: Baloxavir Acid; BID: twice daily; BXM: Baloxavir Marboxil; Cmax: maximum plasma concentration; QD: once daily; LD50:Lethal Dose, 50%; PK: pharmacokinetics; PO: per os (oral administration); RTP: ribofuranosyl 5’-triphosphate; T_max_: time to reach maximum plasma concentration; TTAS: time to alleviation of symptoms. CL/F: apparent oral clearance

**SUPPLEMENTARY TABLE 3 Efficacy Outcomes of RdRp-targeting Inhibitors**

| **Study (Author, Year)** | **Study Type** | **Drug** | **Population** | **Time to Symptom Relief (hours)** | **Virological endpoints** | **Key findings** | **Reference** |
| --- | --- | --- | --- | --- | --- | --- | --- |
| Retout et al., 2022 | Extrapolation study | BXM (40/80 mg) | Asian and non-Asian adults with influenza A/B | Asian: 62.4  Non-Asian: 91.2 | - | Ethnicity (Asian vs. non-Asian) did not significantly impact the drug effect on TTAS; predicted TTAS was similar across different Asian populations. | (Retout et al., 2022) |
| NCT01068912 | Phase 2, randomized, double-blind, placebo-controlled, multicenter study | Favipiravir (Low dose: 1000 mg BID Day 1, then 400 mg BID x 4 days) | Adults with uncomplicated influenza | 100.4 (82.4–119.8) | - | Favipiravir showed a dose dependent anti-influenza activity. | (MDVI, LLC, 2015) |
|  |  | Favipiravir (High dose: 1200 mg BID Day 1, then 800 mg BID x 4 days) |  | 86.5 (79.2–102.1) | - |  |  |
| NCT02949011 (CAPSTONE-1) | Phase 3 | BXM (40/80 mg single dose) | Adults & adolescents with influenza | 53.7 (49.5–58.5) | 24 h (24.0 to 48.0) | BXM was efficacious and safe. | (Shionogi, 2019) |
| Fujita et al., 2020 | Retrospective observational study | BXM (40 mg single dose) | Inpatients during nosocomial outbreak (oseltamivir-resistant A (H1N1)pdm09) | The time to  alleviation of fever (hours) between the oseltamivir (n = 11) and BXM groups (n = 13) was significantly different (p= 0.0034) | - | BXM was effective in controlling nosocomial outbreaks caused by oseltamivir-resistant influenza A(H1N1)pdm09 virus. | (Fujita et al., 2020) |
| Collins C et al., 2022 | Subgroup analysis of CAPSTONE-2 | BXM | Patients aged ≥ 12 years with cardiovascular disease | Median TTIIS in patients with CVD:  BXM: 69.8 h (95% CI 56.4-91.7) oseltamivir: 91.3 h (95% CI 54.5-127.0)  Median TTAS:  BXM :72.4 h (95% CI 56.4-91.7) Oseltamivir: 103.7 h (95% CI 55.1-138.8; p=0.0287) | - | Patients with CVD may derive clinical benefit from prompt BXM treatment, given the shorter median TTIIS and significantly shorter median TTAS vs oseltamivir. | (Collins et al., 2022) |
| Watanabe et al., 2019 | Phase 2, double-blind, multicenter, placebo-controlled study | BXM 10/20/40 mg | Patients aged ≥20 to <65 years | Median TTAS  Influenza A/H1N1pdm  52.9 (10mg)  47.1 (20mg)  48.2 (40 mg)  Influenza B  63.3 (10mg)  65.4 (20mg)  63.3 (40 mg)  Time to treatment >12 to ≤24 h  52.4 (10mg)  63.9 (20mg)  53.5 (40 mg)  Time to resolution of fever  10mg: 33.4 (26.9–38.1)  20mg: 31.6 (26.9–35.8)  40mg: 28.9 (24.5–34.7) | Statistically significant difference in the change from baseline in virus titer for the A/H3N2 virus 48 hpi in the 10 mg and 40 mg (p < 0.05 for 10 mg and 40 mg). For A/H1N1pdm and A/H3N2, virus titer was close to the lower limit of quantification by Day 2 | BXM was effective regardless of virus type/subtype. | (Watanabe et al., 2019) |
| Baba et al., 2020 | Double-blind, placebo- and oseltamivir-controlled, randomized study | BXM | Healthy outpatients with acute uncomplicated influenza | - | Reduction in viral titer 24 h after BXM administration (P<0.0001) | Little impact of BXA carryover on the infectious titer testing. | (Baba et al., 2020) |
| Yoshino et al., 2020 | Prospective Observational Study | BXM 40/80 mg, Oseltamivir 75 mg, Laninamivir 40 mg | Influenza patients | Median recovery for sore throat:  BXM 72  Oseltamivir 72  Laninamivir 36  Nasal discharge:  BXM 60  Oseltamivir 72  Laninamivir 48  Headache:  BXM 48  Oseltamivir 48  Laninamivir 36  Muscle pain:  BXM 36  Oseltamivir 48  Laninamivir 24  Time to defervescence:  BXM 24 (24-48)  Oseltamivir 72 (24-72)  Laninamivir 48 (36-84) | - | BXM has a better antipyretic effect than oseltamivir and laninamivir. | (Yoshino et al., 2020) |
| Jiang et al., 2025 | Multicenter, retrospective, cohort study | BXM 40 mg for those weighing <80 kg (except two patients taking 20 mg) and 80 mg for those weighing over 80 kg | Patients with confirmed influenza (RT-PCR positive, no other infections) | Median TTAS  BXM 96  Oseltamivir 120  Median time for fever resolution:  BXM 72  Oseltamivir 72 | No significant difference in viral clearance | No difference in time to alleviation of symptoms (P = 0.054) or duration of fever (P = 0.347) between BXM and oseltamivir. | (Jiang et al., 2025) |
| Ringer et al., 2024 | Retrospective cohort study | BXM | Immunocompromised adult patients | Median time to fever resolution  21.6 vs 26.6 (BXM vs Oseltamivir) | - | No significant difference in median time to hypoxia/fever resolution. | (Ringer et al., 2024) |
| Shah et al., 2020 | Multicenter, retrospective | BXM | Adult patient with influenza A | Median time to fever resolution  H1 influenza  20.325 (6.938–43.792) vs 33.658 (21.896–49.175) (BXM vs Oseltamivir)  Time to treatment <48 h  25.183 (8.85–40.117) vs 24.075 (10.158–39.175)  (BXM vs Oseltamivir)  Time to hypoxia resolution 51.7 (25.3–89.3) vs 72 (37.5–123) | - | BXM had a significantly faster time to hypoxia resolution (P < 0.001). | (Shah et al., 2020) |
| Shah et al., 2019 | Multicenter, retrospective, chart review | BXM | Adult patient with influenza A | 43 vs 81 (BXM vs Oseltamivir) | - | BXM had possible benefits of reduced length of stay and faster time to resolution of hypoxia compared with oseltamivir. | (Shah et al., 2019) |
| Kumar et al., 2022  (FLAGSTONE) | Randomised, parallel-group, double-blind, placebo-controlled, superiority trial | BXM 40 mg (for 40 kg to <80 kg bodyweight) or 80 mg (for ≥80 kg bodyweight) on day 1 and day 4 | Patients ≥12 yrs, hospitalized with influenza | Median time to clinical improvement: 97.5 h vs 100.2 in control group | Faster reduction in viral load; reduced emergence of resistance | BXM in combination with NAI did not result in superior clinical outcomes than NAI alone. | (Kumar et al., 2022) |
| Zhao et al., 2025 | Phase 2/3 | ADC189 | Adult population | Median time to symptom alleviation 50.0 h (95% CI, 44.6 to 59.3)  PA-I38 substitutions 74.7; PA non-I38 45.7 | Time for viral clearance  50.7 h (15 mg)  45.8 h (45 mg)  By 24 hpi, the decrease in viral load from baseline was greater with ADC189 vs placebo (2.316 and 1.049 log_10_ virus copies per milliliter) | A single-dose ADC189 shortened the time to the resolution of symptoms. | (Zhao et al., 2025) |
| Yoshimura et al., 2020 | Single-center, retrospective observational analysis | BXM + Peramivir | Hospitalized adults with influenza | Median time to defervescence  50.4 (28.8) h (n = 7) and 62.4 (67.2) h (n = 107) | - | BXM combined with peramivir was more effective than peramivir without BXM. | (Yoshimura et al., 2020) |
| Ison et al., 2020 | Randomised, placebo-controlled,  phase 3 trial (CAPSTONE- 2) | BXM vs Oseltamivir | Outpatients aged ≥12 yrs with risk factor for influenza-associated complications | Median TTIIS  BXM 73.2 (95% CI  67.2 to 85.1)  Placebo 102.3 [92.7 to 113.1]; difference 29.1 (95% CI 14.6 to 42.8; p<0.0001)  Oseltamivir 81.0 h (95% CI 69.4 to 91.5  By 24hpi, the mean reduction in virus titer from baseline  BXM 3.36 log_10_TCID_50_/mL Oseltamivir 1.76  Placebo 1.25 | By 24h after initiation of the trial regimen, the mean reduction in virus titer from:  BXM:3·36 log_10_TCID_50_/mL.  Oseltamivir: 1·76 log_10_TCID_50_/mL  The reductions in viral RNA load were faster in BXM group vs oseltamivir and placebo | Single-dose BXM had superior efficacy to placebo and similar efficacy to oseltamivir for ameliorating influenza symptoms in high-risk outpatients. | (Ison et al., 2020) |
| Cagas et al., 2020 | Subgroup Analysis of CAPSTONE- 2 | BXM, Oseltamivir | Patients with CLD | Median TTIIS  BXM 77 (95% CI: 67.4-93.3)  Placebo 113.2 (95% CI: 100.1-137.8)  Oseltamivir 91.2 (95% CI: 69.8-101.4)  Median time to cessation of viral shedding  BXM (48 h)  Placebo (96 h; p<0.0001) Oseltamivir (72 h; <0.0001) | - | Prompt BXM treatment significantly shortens influenza-associated symptoms in patients with CLD. | (Cagas et al., 2022) |
| Yang et al., 2025 | Multicentre, double-blind, randomised, placebo-controlled and oseltamivir-controlled, phase 3 trial | Onradivir, Oseltamivir | Adult patient | Median TTAS  Onradivir 38.83 (95% CI 35.32–41.18)  Oseltamivir 42.17 (95% CI 38.27–52.83)  Placebo 63.35 (95% CI 55.48–68.48) | Decrease in mean viral titer (log_10_ TCID_50_/mL) from baseline after 24 h  onradivir (–0.87 [SE 0.04])  oseltamivir (–0.66 [0.06]  placebo (–0.72 [0.06], p=0.017)  Decrease in viral load (log_10_ copies per mL)  Onradivir –1.22 (SE 0.05)  Oseltamivir 0.70 (0.07)  Placebo –0.69 (0.07, (p<0.0001)) | Onradivir has similar curative efficacy to oseltamivir for acute, uncomplicated influenza infections. | (Yang et al., 2025) |
| Wang et al., 2025 | Multi-center, randomized, 3 double-blind, placebo-controlled phase 2 | Suraxavir (GP681) 40 mg, 20 mg | Adult patients aged 18–65 with uncomplicated influenza B | Median TTAS  40 mg: 50.0 (44.1, 71.9)  20 mg: 46.1 (39.6, 58.2)  Placebo: 82.3 (67.9, 87.3)  Medians time to remission of fever  40 mg: 27.0 (95%CI: 21.70, 34.30)  20 mg: 22.9 (95%CI: 20.00, 31.30)  Placebo: 47.2 hours (95%CI: 34.30, 60.10) | Mean changes in viral titers (log_10_) from baseline  40 mg: -2.14 (1.97)  20 mg: -1.48 (2.01)  Placebo: -0.69 (1.98)  Median viral clearance time  40 mg: 23.5  20 mg: 35.2  Placebo: 45.6 | Single-dose suraxavir was effective in improving clinical symptoms and accelerating viral clearance. | (Wang et al., 2025b) |
| Wang et al., 2025 | Randomized, double-blind, placebo-controlled phase 2/3 trial | ZX-7101A 40/80 mg | Adults with uncomplicated influenza with  fever (axillary temperature ≥37.3 ºC | Phase 2  Median TTAS  40 mg: 34.7 hours (95% CI,  22.8–43.4)  80 mg: 45.8 hours (95% CI, 32.0–66),  Placebo: 63.6 hours (95% CI, 43.9–93.4)  Phase 3  24.1% (165/684) were treated within 24 hours from  symptom onset  Median TTAS  40 mg: 48.4 hours (95% CI, 40.5–55.6)  80 mg: 39.4 (95% CI, 35.8–49.3)  Placebo: 62.9 (95% CI, 56.4–69.3)  Median difference vs placebo of TTAS  Time since symptom onset  ≤ 24 hours  40 mg: 1.83 (95% CI, 1.294–2.585)  80 mg: 2.043 (95%CI, 1.442–2.891)  >24 hours  40 mg: 1.163 (0.926–1.461)  80 mg: 1.336 (1.065–1.678) | Median time to negative detection of viral RNA  40 mg: 43.15 (39.40–44.75)  80 mg: 41.37 (38.63–43.60)  Placebo: 90.73 (87.23–94.56)  Median time to negative detection of viral titer  40 mg: 23.10 (22.85–23.43)  80mg: 22.66 (22.31–23.01)  Placebo: 24.85 (23.96–27.35) | ZX-7101A (single dose of either 40 mg or 80 mg), was effective, with more rapid alleviation of influenza symptoms vs. placebo.  The proportion of patients with detectable influenza virus RNA or virus titer at 24, 48, and 96 h post-treatment significantly decreased with ZX-7101A treatment vs. placebo. | (Wang et al., 2025a) |
| Monto et al., 2025 | Multicountry, phase 3b trial | BXM 40mg/80mg | Influenza-positive index patients aged 5 to 64 years and  household  contacts | - | By 72 hpi the adjusted mean reduction from baseline in viral titer  BXM: 2.22 log_10_ TCID_50_/mL  Placebo: 1.85 log_10_ TCID_50_/mL | BXM resulted in a more rapid reduction in virus titer and viral load in index patients than placebo. | (Monto et al., 2025) |
| Yoshino et al., 2019 | Prospective Observational Study | Oseltamivir, Laninamivir, BXM | Adult patients with seasonal Influenza | Mean time to defervescence  BXM 37.68 (18.24)  Oseltamivir 55.92 (29.52)  Laninamivir 60 (30.24) | - | Regarding the antipyretic effect, BXM was clinically superior to laninamivir (P=0.0231).  No significant difference between the BXM and oseltamivir with respect to the time to defervescence (P= 0.0853). | (Yoshino et al., 2019b) |
| Kawaguchi et al., 2018 | CAPSTONE-1 Study, a phase I, single-center, open-label, randomized, six-sequence, three-period, and three-treatment crossover study. | BXM, Oseltamivir | Patients aged 12–64 with ≤48 hours from flu symptom onset. | Median TTAS reduced by 30.6 hours vs placebo (87.3 vs 117.9)  Time to treatment form symptom onset  BXM:  Early  Median TTAS 49.3 (*N =* 238)  Late  Median TTAS 66. 2 (*N =* 217)  Placebo:  Early Median TTAS 82.1 (N = 120)  Late Median TTAS 79.4 (N = 110) | Time to cessation of viral shedding: 24 vs 72 hours  Time to treatment form symptom onset  Early  Mean Change (Log_10_ [TCID_50_/mL]) of Viral Titer at 24 hpi -4.46 (*n =* 180)  Late  Mean Change (Log_10_ [TCID_50_/mL]) of Viral Titer at 24 hpi -4.32 (*n =* 160) | Early treatment with BXM leads to a significantly faster TTAS vs. placebo and causes significant viral titer reduction regardless of treatment time. | (Kawaguchi et al., 2018a) |
| Li et al., 2023 | Observational study | BXM, Oseltamivir, Laninamivir, Zanamivir | Children and adult patients with influenza-like symptoms | - | Median viral clearance between Influenza A and B:  BXM 0.81/0.77, Laninamivir 0.76/0.37, Oseltamivir 0.63/0.64, Zanamivir 0.90/0.55 | Reduction in daily viral RNA shedding was faster in BXM compared with laninamivir and oseltamivir. | (Li et al., 2023) |
| Hayden et al., 2022 | Two Phase 3, Randomized, Double-Blind, Placebo-Controlled Trials | Favipiravir | Otherwise healthy adults with influenza-like symptoms and fever of ≤48 hours | Median time to illness alleviation  Favipiravir 84.2 (US316) Placebo 98.6  Favipiravir 77.8 (US317)  Placebo 83.9  Time from onset to first dose  US316  <24 h: 95.7 (77.6–106.9)  ≥24 h: 79.0 (72.2–95.6)  US317  <24 h: 79.0 (71.8–94.4)  ≥24 h: 76.0 (71.8–82.0) | Favipiravir was associated with reduced viral titers, RNA load area under the curve over 24–120 h, and median times to cessation of virus detection (*P*<.001).  Viral titer change baseline to 72 hpi,  log_10_ TCID_50_/mL, mean (SD)  US316: -2.1 (1.7)  US317: -2.6 (1.9) | Favipiravir dosing regimen demonstrated significant antiviral efficacy but inconsistent illness alleviation in uncomplicated influenza. | (Hayden et al., 2022) |
| Trevejo et al., 2018 | Phase IIa, randomized, double-blind, placebo-controlled study | Pimodivir | Adult volunteers experimentally inoculated with live influenza virus | - | All doses reduced the magnitude of viral shedding.  Viral shedding duration (log_10_ TCID_50_/mL*day):  Pimodivir_100 mg: 1.25 (0.0-16.1)  Pimodivir_400 mg: 0.70 (0.0-18.0)  Pimodivir_900/600 mg: 3.20 (0.0-16.1)  Pimodivir_1,200/600: 0.35 (0.0-8.4)  mg  Pooled:0.65 (0.0-18.0 | All doses of pimodivir except 900/600 mg reduced duration of viral shedding | (Trevejo et al., 2018) |
| NCT1068912 and NCT01728753 | Post-hoc analysis of 2 Phase 3 trials | Favipiravir | Adults with uncomplicated influenza | - | Approximate 0.3–0.4 log_10_ TCID_50_/mL greater reductions within 48 h in those with favipiravir Cmin ≥20 μg/ mL compared to those with lower concentrations | Dose dependent antiviral effects and more rapid illness alleviation. | (Hayden et al., 2024) |
| Ison et al., 2021 | Subgroup Analysis of the Phase 3 CAPSTONE-2 Trial | BXM, Oseltamivir | Influenza patients with diabetes | Median TTIIS  BXM vs oseltamivir vs placebo 75.4 vs 76.5 vs 125.0 | Median time-to-viral-clearance  BXM vs oseltamivir vs placebo 48 hours vs 96 hours vs 96 hours | Clinical and virological efficacy was observed. | (Ison et al., 2021) |
| Liu et al., 2022 | Randomized, open-label, phase I study | BXM | Healthy Chinese patients 20–59 yrs | Simulated TTAS  Otherwise healthy patients  BXM 57.1 (54.2–60.5)  Placebo 75.4 (70.8–79.6)  High risk  BXM 65.2 (61.3–69.0)  Placebo 75.4 86.4 (81.7–90.4) | - | Simulated median TTAS for Chinese patients agreed with simulated values in Asian patients. | (Liu et al., 2022) |
| Finberg et al., 2019 | Phase 2b, randomized, double-blinded, placebo-controlled, parallel-group, dose-response, multicenter study | Pimodivir 300 mg twice daily  Pimodivir 600 mg twice daily  Pimodivir 600 mg + Oseltamivir 75 mg twice daily | Adults aged 18–64 years with influenza-like illness, fever ≥38°C, symptoms ≤48 hours, and influenza A–positive test, without major comorbidities. | Faster time to resolution of influenza symptoms versus placebo observed with pimodivir 600 mg (13%) and pimodivir 600 mg plus oseltamivir 75 mg (17%). | The average changes in the virus load AUCs (TCID50)/mL from that of the placebo group:  Pimodivir 300 mg twice daily: –2.1 (–2.9 to –1.3)  Pimodivir 600 mg twice daily: –2.1 (–2.8 to –1.3)  Pimodivir 600 mg + Oseltamivir 75 mg twice daily: –2.0 (–2.8 to –1.2) | Pimodivir (with or without oseltamivir) resulted in significant virologic improvements over placebo, demonstrated trends in clinical improvement and was well tolerated. | (Finberg et al., 2019) |
| Wang et al., 2020 | Phase 2a, open-label, dose-escalating, multi-center pharmacokinetic study | Favipiravir | Critically ill adult hospitalized patients (aged≥18 years with influenza A or B and respiratory failure | - | Mean viral RNA load AUC measured from baseline to 120 hpi was lower for the 1800/800 mg groups than the 1600/600 mg group (p = 0.0032) | The two dosing regimens proposed for uncomplicated influenza did not achieve C_trough_ ≥20 mg/L. | (Wang et al., 2020b) |
| Koshimichi et al., 2020 | Population pharmacokinetics study | BXM, Oseltamivir | Healthy subjects / otherwise healthy/ at high risk of influenza complications | - | Influenza A: Change from baseline in virus titer (log_10_ TCID_50_/mL) on day 2:  BXM (≥20 ng/mL):-3.8 to -4.5  Placebo: -1.4  Oseltamivir -2.3  Influenza B:  BXM (≥20 ng/mL):-2.3 to -4.0  Placebo: -0.8  Oseltamivir -1.0 | Body weight-based dosing was reported to be effective across influenza types. | (Koshimichi et al., 2020) |
| Liao et al., 2023 | Prospective observational study | BXM (40 or 80 mg) | Adults and  adolescents with uncomplicated  influenza from China | Duration of fever in BXM treated patients was  1.5 (1.0-2.5) days vs 2.5 (1.5–3.0) days in oseltamivir group | - | BXM demonstrated comparable clinical benefits to oseltamivir | (Liao et al., 2023) |
| Cai et al., 2024 | Ambispective, observational, multi-center study | BXM | Outpatients with uncomplicated influenza | Median TTAIS:  BXM 28 (IQR 20–50)  Oseltamivir 48 (IQR 30–67)  Median TTAF: BXM 18 (IQR 10–24), Oseltamivir 30 (IQR 19–48)  Hazard Ratios: TTAIS HR = 1.36, TTAF HR = 1.93 (BXM vs. Oseltamivir). | - | BXM was superior to Oseltamivir in alleviating influenza symptoms in outpatients with uncomplicated influenza. | (Cai et al., 2024) |
| Yang et al., 2024 | Multicentre, doubleblind, randomised, placebo-controlled, phase 2 trial | Onradivir | Adults with acute uncomplicated influenza | Median time to alleviate influenza symptoms  200 mg BID 46.92 (IQR 24.00–81.38)  400 mg BID 54.87 (23.67–110.62)  600 mg 40.05 hours (17.70–65.82)  placebo group 62.87 (36.40–113.25). | The mean difference in virus load AUC from the baseline to 144 hpi  Onradivir 200 mg: 52.24 h × log_10_ copies per mL (95% CI 0.56–3.97)  Onradivir 400 mg: 42.39 h × log_10_ copies per mL (0.02–3.51)  Onradivir 600 mg 57.33 h × log_10_ copies per mL  (0.7–4.07)  Decrease in viral load  24 h after initiation of onradivir treatment  Onradivir 200 mg: 1.92 (95% CI 1.51–2.32) log_10_ copies per mL  Onradivir 400 mg: 1.63 (1.22–2.04) log_10_ copies per mL  Onradivir 600 mg: 2.04 (1.64–2.44) log_10_ copies per mL    Placebo: 0.64 log_10_ copies per mL (0.24–1.05) | Onradivir showed higher efficacy than placebo in ameliorating influenza symptoms and lowering the viral load. | (Yang et al., 2024) |
| Wang et al., 2025 | Multicenter, randomized, double-blind, placebo-controlled phase 3 trial | Suraxavir (GP681) | Outpatients aged 5–65 yrs with uncomplicated influenza | Median TTAS was significantly shorter in the suraxavir 42.0  Placebo 63.0  Median difference: −20.3 (95% CI 11.60–23.76) | Rapid decreases in viral load from baseline than placebo by day 1 after initiation of the trial regimen (*P* < 0.001); the mean (SD) changes in viral load were −2.2 (1.3) log_10_ copies per mL in the suraxavir group and −1.3 (1.7) log_10_ copies per mL in the placebo group.  On day 2, the mean (±s.d.) changes from baseline in the H1N1pdm group and H3N2 group  were −2.4 (1.2) and −2.1 (1.4) log_10_ copies per mL, respectively  Viral load changes in children, adolescents and adults (−2.4 (1.3) versus −2.1 (1.2) log_10_ copies per mL) in the first 24 h.  Median time to viral clearance with suraxavir vs placebo (22.0 h vs 46.9 h, *P* < 0.001; 95% CI = 4.48-7.44) | Timely single-dose suraxavir marboxil was effective in shortening TTAS and reducing the influenza viral load. | (Wang et al., 2025c) |
| Qiu et al., 2024 | Prospective, randomized and parallel-controlled trial | BXM and Oseltamivir | Patients with confirmed influenza | BXA  Fever 36.96 (15.85)  Cough 54.24 (21.84)  Sore throat 49.44 (20.64)  Oseltamivir  Fever 40.08 (17.04)  Cough 55.2 (21.6)  Sore throat 50.16 (19.92) | - | There was no significant difference in fever subsidence time cough improvement time and sore throat improvement time between the two groups (all *p* > 0.05)  BXM can be effectively used in the treatment of patients with influenza A. | (Qiu et al., 2024) |
| Yoshii et al., 2020 | Multicenter, observational study | BXM and NAIs | Patients prescribed anti-influenza drug | Mean time to the alleviation of the fever 46.56 (2.16)  Rate of alleviation of the fever  within 1 day from the start of anti-influenza drugs between  the groups with times from the onset to the start of antiinfluenza drugs≤2 days and ≥2.5 days; the results were 3.8% (10/270), and 20.8% (5/24), respectively (p=0.003). | - | BXM was more effective than NAI, with a significantly shorter duration of the fever. | (Yoshii et al., 2020) |
| Ushara et al., 2020 | CAPSTONE-1 Phase 3, double-blind, placebo- and active  comparator-controlled, randomized trial | BXM | Adult patients | The median times to alleviation of symptoms were 63.1, 51.0, and 80.2 hours, in the BXM recipients with PA/I38X-substituted viruses, without PA/I38X-substituted viruses, and placebo recipients, respectively | Median time to sustained cessation of infectious virus detection was 192, 48, and 96 hours in the BXM recipients with PA/I38X-substituted viruses, without PA/I38X-substituted viruses, and placebo recipients, respectively | The emergence of viruses with PA/I38X substitutions following BXM treatment was associated with transient rises in infectious virus titers, prolongation of virus detectability, initial delay in symptom alleviation, and uncommonly with symptom rebound. | (Uehara et al., 2020) |
| Goto et al., 2024 | Non-randomized, prospective, observational study | BXM | Outpatients diagnosed with A(H3N2) influenza | The median durations of fever for patients infected with influenza A(H3N2) in the baloxavir, oseltamivir, and other NAI groups were 27.0 h (95% CI, 23.0–37.0), 38.0 h (95% CI, 30.5–41.5), and 36.0 h (95% CI, 24.0–45.0), respectively. | BXM: viral detection was 11.1% (4/36) and 0% (0/36) at 120 hpi and 240 hpi, respectively. | BXM’s virological and clinical effectiveness against A(H3N2) | (Goto et al., 2024) |
| Chong et al., 2021 | Non-randomized, prospective, observational study | BXM | Outpatients diagnosed with influenza | Median duration of fever: 22.3 h (95% CI, 20.5–25.5)  Median duration of symptoms: 62.0 h (95% CI, 52.1–75.5) | The isolation frequencies of A(H1N1)pdm09 at 120 h and 240 h after  BXM treatment were 5.2% (3/58) and 0.0% (0/58), respectively | BXM was virologically effective for influenza | (Chong et al., 2021) |
| Fang et al., 2024 | Multicenter, Randomized, Placebo-Controlled Phase III Trial | TG-1000 | Adolescent and Adult Outpatients with Acute Uncomplicated Influenza | The median alleviation time was shorter in the TG-1000 group compared to the placebo group (60·9 h vs. 87·9 h, HR = 1·683 [95% CI:1·435, 1·975], p < 0·0001) | Time to negative virus RNA status of TG-1000 vs placebo:  91·6 h (89·8, 93·6) vs. 95·9 h (93·0, 112·7), p = 0·0008  Time to negative titer: 22·3 h (21·5, 22·7) vs. 39·9 h (35·2, 42·5), p < 0·0001 | Single-dose of TG-1000 was associated with noticeable clinical benefit and antiviral activity. | ((Fang et al., 2024) |

AEs: Adverse Event; BID: Twice a day; BXA: Baloxavir Acid; BXM: Baloxavir Marboxil; CVD: Cardiovascular Disease; FAERS: Food and Drug Administration Adverse Event Reporting System; FluSurv-NET: Influenza Hospitalization Surveillance Network; ICSRs: Individual Case Safety Reports; RT-PCR: Reverse Transcription Polymerase Chain Reaction; SAEs: Serious Adverse Event; TEAEs: Treatment Emergent Adverse Events; TTAF: Treatment to Alleviation of Fever; TTAS: Time to Alleviation of Symptoms.

Note: Virological endpoints included viral RNA load, infectious viral titer, viral shedding, or RNA-negative conversion depending on the original study. Viral RNA load and infectious viral titer are distinct virological endpoints and were extracted as reported in the original studies.

**SUPPLEMENTARY TABLE 4 Safety Profiles of RdRp-targeting Inhibitors**

| **Drug** | **Population** | **Common Adverse Events (AEs)** | **Serious AEs** | **Discontinuation (All-cause / AE-related)** | **Drug-Drug Interactions / Dose Adjustments** | **Reference** |
| --- | --- | --- | --- | --- | --- | --- |
| BXM | Healthy adults inoculated with influenza A/California/04/2009/H1N1pdm-like  virus | Unsolicited AE: 46 (60.5%)  (most common unsolicited AE: lymphadenopathy 10 [13.2%])  At least 1 challenge-related unsolicited AE: 26 (34.2%)  Challenge-related unsolicited AEs of mild severity: 24 (31.6%)  Challenge-related unsolicited AEs of moderate severity (ear pain, pyrexia): 2 (2.6%) | NR | NR | NR | (Ortiz et al., 2023) |
|  | Healthy Patients with Influenza | Adverse events:20.7  Total:47/610 (7.70%)  Diarrhoea :18/610 (2.95%)  Nausea:8/610 (1.31%)  Bronchitis:16/610 (2.62%)  Sinusitis:7/610 (1.15%) | Total (overall):0.3%  BXM: Incarcerated inguinal hernia:1/610 (0.16%)  BXM: Meningitis viral:1/610 (0.16%) | Overall :0.3% |  | (Shionogi, 2019) |
|  | FAERS database | Number of case reports:  Off-label use: 378  Intentional product use issue: 278  Diarrhoea: 96  Pneumonia: 90  Vomiting: 77  Loss of consciousness: 36  Urticaria: 35  Anaphylactic reaction: 35  Rhabdomyolysis: 25  Seizure: 25  1727 ADE reports were retrieved for BXM, reporting a strong association with ischaemic colitis, melaena, delirium febrile, enterocolitis, febrile convulsion, and altered state of consciousness.  Most cases of anaphylaxis in patients aged 20-59 years. Signals detected were:  ROR: 3.05 (2.22-4.18), IC: 1.44 (0.98-1.90). | NR | NR | NR | (Zhou et al., 2024) (Tanaka et al., 2021) (Tu et al., 2025) |
|  | Household contacts of influenza patients | Any AE 22.2%, nasopharyngitis 6.4%, headache 2.1%, ALT/AST increases 0.3% | NR | NR | Single-dose regimen | (Ikematsu et al., 2020a) |
|  | Adult outpatients with seasonal influenza | Diarrhea 25–46%, nausea/vomiting 7–36% | NR | NR | Weight-based single dose (40/80 mg) | (Yoshino et al., 2020) |
|  | Hospitalized patients with severe influenza (FLAGSTONE) | Any AE 45%, AE with fatal outcome 2%, related AE 3% | 12–15% | AE leading to withdrawal 1–3% | NR | (Kumar et al., 2022) |
|  | Outpatients diagnosed with influenza | Incidence of bleeding for BXM was similar to that for other anti-influenza treatments (odds ratios: 0.90–0.99) | NR | NR | NR | (Hara et al., 2022) |
|  | High-risk adolescents/adults (CAPSTONE-2) | Any AE 25–30%, bronchitis 3–5%, sinusitis 2–3%, diarrhea/nausea 3–5% | 1% | NR | NR | (Ison et al., 2020) |
|  | Healthy Subjects | Any TEAEs: 2 (11.8)  Infections and infestations: 1 (5.9)  Viral infection: 1 (5.9)  Investigations: 1 (5.9)  Aspartate aminotransferase increased: 1 (5.9)  Blood lactate dehydrogenase increased: 1 (5.9) | NR | NR | No clinically meaningful drug–drug interaction between BXM and oseltamivir | (Kawaguchi et al., 2018b) |
|  | Healthy Subjects | Six TEAEs were reported by 6 (20%) of the 30 subjects.  Among the TEAEs, two adverse drug reactions (dyspepsia, 20 mg dose group; headache, 80 mg dose group [≥80 kg]) were observed | NR | NR | NR | (Kim et al., 2022) |
|  | Healthy Chinese adults aged 20–59 years with BMI 18.5–<26 kg/m² and Chinese ancestry. | AEs:  40 mg group: 3  80 mg: 16  AEs reported in more than one individual  across both dosing cohorts included dizziness (*n* =3), upper respiratory tract infection (*n* = 3), blood uric acid increased (*n* = 3), blood bilirubin increased (*n* = 3), and diarrhea (*n* = 2). | 0 | NR | NR | (Liu et al., 2022) |
|  | Adult outpatients with uncomplicated influenza A | Mild AEs: bronchitis, rash, asthma (0.91%) | NR | NR | NR | (Cai et al., 2024) |
|  | Healthy adults | TEAE:  BXM 6mg: 1/6 (16.7%)  BXM 20 mg:  Fasted subjects: 7/15 (46.7%)  Fed subjects: 3/12 (25%)  Before meals: 1/12 (8.3%)  Most common TEAEs: headache, increased ALT, increased eosinophil count, increased WBC | 0 | 3/15 (20.0%) in fasted subjects with 20 mg BXM | NR | (Koshimichi et al., 2018) |
|  | Adults with influenza A | Most common AEs: nausea/loss of appetite 3–6%, diarrhea 2–3%, dizziness/headache 2–4%, rash 1% | NR | NR | NR | (Qiu et al., 2024) |
|  | | | | | | |
| Favipiravir | Adults with uncomplicated influenza | TEAE 25–28%, treatment-related 7–10%  Headache 2–5%, diarrhea 2–5%, nausea 1–5%, ALT/AST/creatinine/fibrinogen abnormalities 1–3% | 0.5–0.7% | 0.5–0.7% | No dose adjustments required (547)  Fixed oral dose: 1800 mg BID Day 1, then 800 mg BID Days 2–5 (56, 547) | (Hayden et al., 2022) (MDVI, LLC, 2015) |
|  | Adults with severe influenza | NR | 60% of patients experienced SAE.  The most  common SAE were secondary bacterial infection (31.4%) and acute liver  injury (11.4%) | NR | Dose escalation: 1600/600 mg BID vs 1800/800 mg BID | (Wang et al., 2020b) |
|  | EudraVigilance database | 157 individual case safety reports  were identified, accounting for 332 ADRs. | Serious cases accounted for 86.4% of  ADRs, with the most affected organ systems including  hepatic, renal, cardiovascular, and nervous systems. | - | - | (Faculty of Medicine and Pharmacy, University of Oradea, Oradea, Romania et al., 2025) |
|  | | | | | | |
| Onradivir (ZSP1273) | Healthy adults aged 18–45 years with BMI 18–26 kg/m² and no significant medical conditions. | Drug related adverse events: (11/36, 30.6%) diarrhea:5/36(13.9%)  increased blood triglycerides:4/36 (11.1%) | NR | NR | No clinically significant drug–drug interaction was observed with the combination of ZSP1273 and oseltamivir | (Pang et al., 2025) |
|  | Adults with acute uncomplicated influenza A | Any AE 49-74%,  Most common (>5%):  diarrhea 33-65%, nausea/vomiting 2–10%, dizziness 2–5%, other GI 1–5% | 0–1% | NR | NR | (Yang et al., 2025) (Yang et al., 2024) (Li et al., 2025a) |
|  | Participants with severe renal impairment vs healthy controls | TEAE 37–75%, cardiac: sinus bradycardia/AV block, renal impairment, GI: diarrhea/abdominal pain | NR | NR | NR | (Li et al., 2025b) |
|  | Healthy Chinese male and female adults aged 18–50 years with BMI 18–28 kg/m² | At least one drug-related AE  Single ascending dose study: 7/43 (16.28%) treated with different doses of ZSP1273.  Multiple ascending dose study: 8/24 (33.33%)  The most frequent drug-related AE (>15%) included diarrhea (37.50–75.00%), leukopenia (18.75%), and neutropenia (18.76%) | NR | NR | NR | (Hu et al., 2021) |
|  | | | | | | |
| Deunoxavir marboxil (ADC189) | Adults/adolescents with uncomplicated influenza | Any AE 35%, nausea 3–4%, diarrhea 3–4%, leukopenia 1%, hypertriglyceridemia 4% | 0–0.5% | AE leading to discontinuation 0–0.2% | NR | (Zhao et al., 2025) (Wei et al., 2025) |
|  | | | | | | |
| Suraxavir Marboxil (GP681) | Adults with acute uncomplicated influenza | Any AE 41-47%, drug-related 39%, Grade 1–2 AE 31–36%  max Grade 3 AE 5%, max Grade 4/5 AE 0–0.3%, labs: triglycerides, neutrophil count | 0 | NR | NR | (Wang et al., 2025b), (Han et al., 2024), (Wang et al., 2025c) |
|  | | | | | | |
| Sebaloxavir marboxil (ZX-7101A) | Adults with uncomplicated influenza | TEAE 41–53%, treatment-related 7–9%, Grade >3 4–7%, neutrophil/WBC decrease 0.8–4.6%, ALT/AST increase ≤0.8% | 0.4% | 0–0.4% | NR | (Wang et al., 2025a) |
|  | Healthy Chinese adults | TEAE ≥1 in 34% overall; system-specific: lab, GI, musculoskeletal, respiratory 2–22% | NR | NR | NR | (Wu et al., 2025) |
|  | | | | | | |
| Pixavir marboxil (TG-1000) | Healthy Chinese adults | Any AE 25–50%, system-organ: urine leukocytes, hyperuricemia, bile acid, CRP, liver labs, ECG abnormalities | NR | NR | NR | (Xu et al., 2023) |
|  | Adolescent and Adult Outpatients with Acute Uncomplicated Influenza | AEs: 25·0% (125/500) vs 28·8% in placebo.  The most common AE with an incidence of ≥ 1.0%: decreased neutrophils count, hyperuricemia, sinus arrhythmia, and vomiting, etc.  One TG-1000 recipient reported a regimen-related AE of severity ≥ 3. | NR | NR | NR | (Fang et al., 2024) |
|  | | | | | | |
| Pimodivir | Healthy adults inoculated with live influenza virus | Incidence of AE: 9 incidences in 5 patients  Decrease in blood phosphorus: 18.1%  Abnormal spirometry: 13.9%  Influenza-like illness: 34/72 (42.2%)  Increased ALT: 13.9% | NR | NR | NR | (Trevejo et al., 2018) |
|  | Adults 18–64 years with influenza A, symptom onset ≤48 h, fever ≥38 °C, ≥1 respiratory and systemic symptom; major comorbidities, recent vaccination, or relevant drug allergies excluded. | TEAE:  300 mg BID: 32 (43.2)  600 mg BID: 39 (52.7)  Most common AE>5%: Diarrhea 5 (6.8) in 300 mg BID group and 20 (27.0) in 600 mg BID group. | 1 (1.4%) Serious TEAE in 600 mg BID | 300 mg BID: 7 (9.5)  600 mg BID: 3 (4.1) | No apparent drug-drug interaction was observed between pimodivir and oseltamivir | (Finberg et al., 2019) |

AST: aspartate transaminase; ALT: alanine transaminase ;AUC: Area Under the Curve; BXA: Baloxavir Acid; BID, twice a day; BXM: Baloxavir Marboxil; EC₅₀: Half-Maximal Effective Concentration; mAb: Monoclonal Antibody; NAI: Neuraminidase Inhibitor; NR: Not Reported; PBS: Phosphate-Buffered Saline; PFU: Plaque-Forming Units; QD: Once Daily; RNA: Ribonucleic Acid; RT-PCR: Reverse Transcription Polymerase Chain Reaction; TCID₅₀: Tissue Culture Infectious Dose 50%; TTAS: Time to Alleviation of Symptoms; WT: Wild-Type Virus.

**SUPPLEMENTARY TABLE 5 Resistance Mutations and Their Impact on RdRp-targeting Inhibitor Efficacy**

| **Inhibitor** | **Subunit Targeted** | **Resistance Mutation** | **Impact on Drug Activity** | **Reported Frequency** | **Reference** |
| --- | --- | --- | --- | --- | --- |
| BXM | PA subunit | I38T/F/M/N/S/R/V/L, E23K/G, A37T, E199G, E199K | BXM: reduced efficacy; I38T most impactful (∼30–35× EC₉₀ increase vs WT)  ↓ binding affinity; 18× increase in Ki; ↓ inhibition potency  5.0–5.2-fold increase in BXM EC_50_ values in PA E199K mutants | BXM: 2.2% (H1N1), 6.2-9.7% (H3N2); Rare in 216/17 & 2017/18 U.S. isolates; rare in B  Common (I38T/F/M). 5%  I38T/M/F in 2.7%-9.7%, E23K in 1.3% of treated cases; no confirmed household transmission  Detected in 9.7% of treated patients.  PA variants were detected in clinical (1.7%, 1/58; 3.8%, 1/26 for children under 12 years) and isolated (3.4%, 2/58; 3.8%, 1/26 for children under 12 years) samples obtained on day 5 after BXM treatment, but not on day 10. | (Ren et al., 2024), (Gubareva et al., 2019),(Acocal-Juárez et al., 2024), (Fukao et al., 2019b), (Kumar et al., 2022), (Stannard et al., 2022), (Kiso et al., 2018), (Miyazawa et al., 2022), (Suzuki et al., 2021), (Fujii et al., 2025), (Lee et al., 2021), (Guo et al., 2024), (Hashimoto et al., 2021), (Ikematsu et al., 2020b), (Ison et al., 2020), (Umemura et al., 2020), (Takashita et al., 2025), (Hamza et al., 2021), (Noshi et al., 2018), (Yoshino et al., 2019a), (Oh et al., 2023), (Todd et al., 2021),(Hickerson et al., 2022), (Qiu et al., 2024), (Kohlbrand et al., 2024), (Kumar et al., 2021), (Taniguchi et al., 2024)  (Chong et al., 2021) |
|  |  | PA-I38X | Reduced susceptibility to BXM | Emerged in 7.2% of treated index patients; none in household contacts | (Monto et al., 2025), (Jones et al., 2021) |
|  |  | PA-E199D | Reduced susceptibility to BXM (almost three-fold reduction in the median EC_50_ value was observed for H9N2 viruses with the E199D mutation (0.7–1.2 nM for E119D compared to 0.1–0.5 nM for WT) | Found in 8 viruses | (Atim et al., 2022) |
|  |  | E23R | Increases BXM EC_50_ values (13- to 19-fold vs. wild-type), synergizes with PA I38T, and only modestly decreases viral fitness. | Lab-generated | (Jones et al., 2022) |
| Favipiravir | PB1 (near NTP-binding region) | K229R  R292K | Confers resistance; reduces favipiravir incorporation  Favipiravir efficiently inhibited both the variant and WT | Lab-generated | (Komeno et al., 2022), (Goldhill et al., 2021)  (Zhang et al., 2014) |
|  |  | H275Y | Treatment of immunosuppressed mice with high (50 mg/kg) but not low (20 mg/kg) doses of favipiravir in combination with oseltamivir (20 mg/kg) significantly delayed mortality and reduced lung viral titers compared to treatment with a single drug regimen with oseltamivir but did not prevent the emergence of oseltamivir-resistant H275Y neuraminidase variants.  Combination of oseltamivir and favipiravir produced a synergistic improvement in survival rate.  All of the dual H275Y mutant viruses tested were susceptible to BXM and favipiravir | NR | (Baz et al., 2018), (Wang et al., 2020a), (Sleeman et al., 2010)  (Smee et al., 2013), (Ormond et al., 2017), (Mu et al., 2023)  (Takashita et al., 2020) |
|  |  | Transition mutations (C→U, G→A) | Increased mutational load; antiviral effect via lethal mutagenesis | Not mutation-specific; bias observed across genome | (Goldhill et al., 2019) |
|  |  | PB1 V43I | Decreased sensitivity to favipiravir and ribavirin; EC_50_ values increased: | NR | (Cheung et al., 2014) |
|  |  | NR | No substitutions associated  with resistance to oseltamivir or T-705 were detected in influenza A(H5N1) virus Infection in mice.  The susceptibility of influenza B viruses isolated from T-705-treated mice remained comparable to that of viruses from untreated control animals | NR | (Marathe et al., 2016), (Pascua et al., 2019) |
|  |  | HGPRT-deficient MDCK cells | T-705 was totally devoid of antiviral activity | Lab generated | (Naesens et al., 2013) |
|  |  | NR | Absence of phenotypically  resistant viruses after multiple rounds of replication *in*  *vitro* with T-705, indicating that the selection of drug specific  resistance mutations are unlikely *in vitro* | Lab generated | (Baranovich et al., 2013) |
|  |  | VN1203- H274Y and -N294S | T-705 was reported to provide effective protection to mice from lethal infection with oseltamivir-sensitive or -resistant highly pathogenic H5N1 viruses. | Lab generated | (Kiso et al., 2010) |
|  |  | PA-P653L | K229R → resistance; P653L → restores polymerase activity and fitness | NR | (Goldhill et al., 2018), (Goldhill et al., 2021) |
|  |  | A(H1N1)pdm09 viruses possessing NA-R152K, PA-I38T or both mutations | Favipiravir treatment was reported to protect mice from lethal infection with all viruses tested | NR | (Kiso et al., 2023) |
| Novel 1,3-oxathiolane nucleoside derivatives of favipiravir |  | - | Significant resistance of the influenza A H1N1 strain against the novel compound compared with its sensitivity to T-705 | NR | (Han et al., 2018) |
| Sebaloxavir marboxil (ZX-7101A) | PA subunit | I38T substitution | Reduced susceptibility | 1.8% (5/278 patients) | (Wang et al., 2025a) |
|  |  | E18G (in vitro) | PA-E18G was identified after serial passage in vitro; it reduced susceptibility to ZX-7101* and BXA. | Observed in influenza A strains in vitro but its clinical relevance remains unknown. | (Luo et al., 2023) |
| Suraxavir marboxil (GP681) | PA subunit | I38T | Associated with reduced antiviral efficacy | H1N1pdm: 0.7% (1/138); H3N2: 0.9% (2/213).  Low PA-I38T rates were reported for GP681, but the denominator definition should be interpreted cautiously. | (Wang et al., 2025c) |
| Pixavir marboxil (TG-1000) | PA subunit | I38X, I38T | Increase in resistance  I38T in 2 patients: 𝐸𝐶_50_ changed from 0·71~0·93 nM before treatment to 73·28~115·1 nM after treatment,  I38T/I in 2 patients: 𝐸𝐶50 from 0·44~0·74 nM to 145·1~184·1 nM, and I38M in 1 patient 𝐸𝐶_50_ from 0·20 to 13·29 nM. | 4·6% (5/108) patients showed I38X-substitution. |  |

EC_50_: Half Maximal Effective Concentration; NA: Neuraminidase; NAIs: Neuraminidase Inhibitors; NTP: Nucleoside Triphosphate; NR: not reported; PA: Polymerase Acidic; PB1: Polymerase Basic Protein 1; RdRP: RNA-dependent RNA polymerase; RNA: Ribonucleic Acid; WT: Wild Type.

*Note: ZX-7101A refers to sebaloxavir marboxil and ZX-7101 refers to its active metabolite.


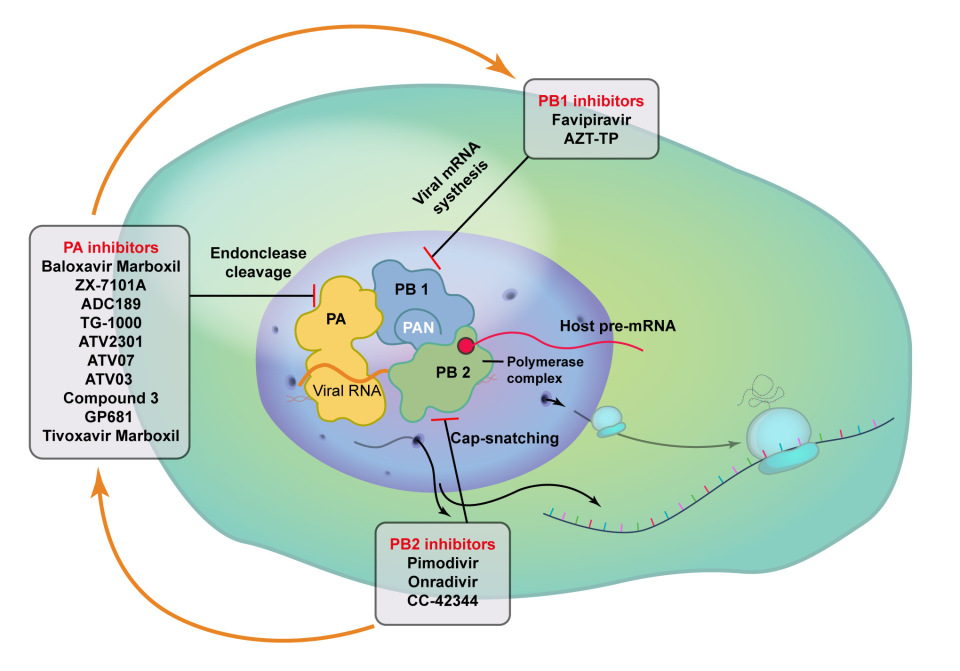


**Supplementary Figure 1. Influenza viral polymerase complex and subunit-specific targets of antiviral inhibitors. PA, PB1, and PB2 inhibitors act on distinct components of the polymerase complex involved in viral RNA transcription and replication**

PA: polymerase acidic protein; PB1: polymerase basic protein 1; PB2: polymerase basic protein 2; PAN: N-terminal domain of the PA protein; RNA: ribonucleic acid; Orange arrows indicate the conceptual grouping of inhibitor classes targeting different subunits of the viral polymerase complex.


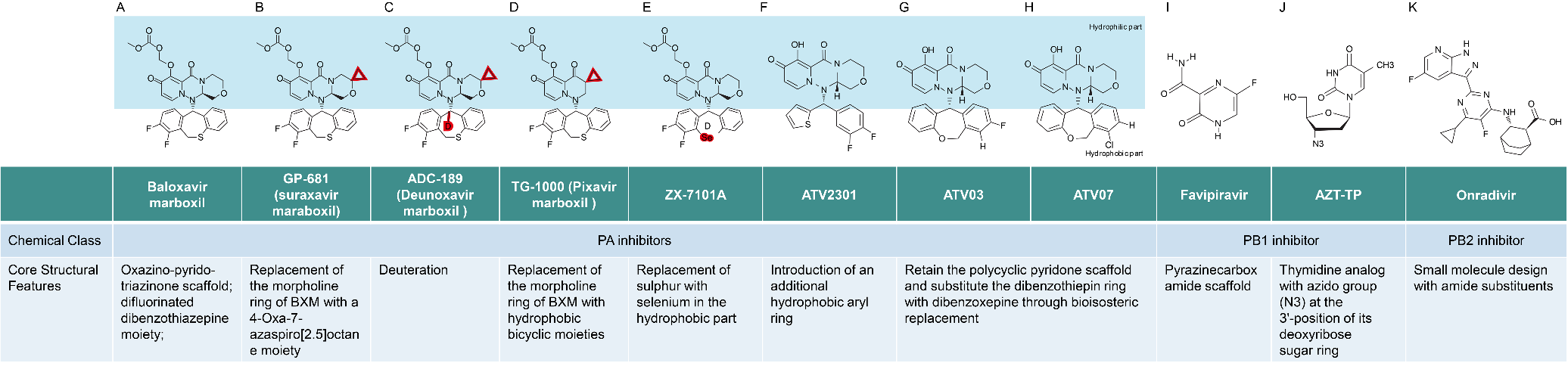


**Supplementary Figure 2. Chemical Structure of Various RdRp-targeting Inhibitors** (**A**) BXM (**B**) Suraxavir marboxil (GP681) (**C**) Deunoxavir marboxil (ADC-189) (**D**) Pixavir marboxil (TG-1000) (**E**) Sebaloxavir marboxil (ZX‑7101A) (**F**) ATV2301 (**G**) ATV03 (**H**) ATV07 (**I**) Favipiravir (J) AZT-TP (**K**) Onradivir (ZSP1273)
